# Correction: Low incidence of antibiotic-resistant bacteria in south-east Sweden: An epidemiologic study on 9268 cases of bloodstream infection

**DOI:** 10.1371/journal.pone.0234276

**Published:** 2020-05-29

**Authors:** The PLOS ONE Staff

The last two authors, Håkan Hanberger and Åse Östholm Balkhed, should be noted as contributing equally to this work. The publisher apologizes for the error.
