# Supplementary material for: Low incidence of antibiotic-resistant bacteria in south-east Sweden: An epidemiologic study on 9268 cases of bloodstream infection
Source: PLoS One. 2020 Mar 27;15(3):e0230501. doi: 10.1371/journal.pone.0230501 (PMC7100936; doi:10.1371/journal.pone.0230501)
Supplement: S1 Fig — (PDF) [file pone.0230501.s001.pdf]

S1 Fig. Bloodstream infections per 100 000 hospital admissions and year.

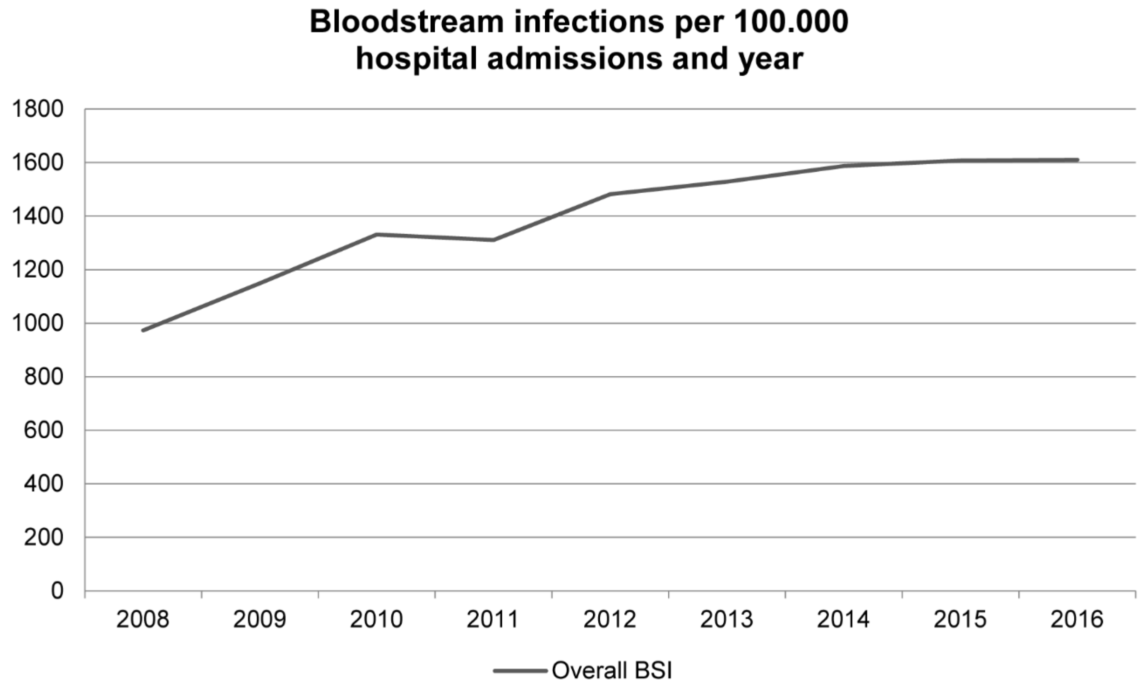

Incidence of BSI increased by 66% from 973 to 1610 per 100 000 hospital admissions per year during the study period (Linear regression) (p=<0.01).
