# Supplementary material for: Low incidence of antibiotic-resistant bacteria in south-east Sweden: An epidemiologic study on 9268 cases of bloodstream infection
Source: PLoS One. 2020 Mar 27;15(3):e0230501. doi: 10.1371/journal.pone.0230501 (PMC7100936; doi:10.1371/journal.pone.0230501)
Supplement: S2 Fig — (PDF) [file pone.0230501.s002.pdf]

S2 Fig. 30-day all-cause mortality due to BSI per 100 000 hospital admissions and year.

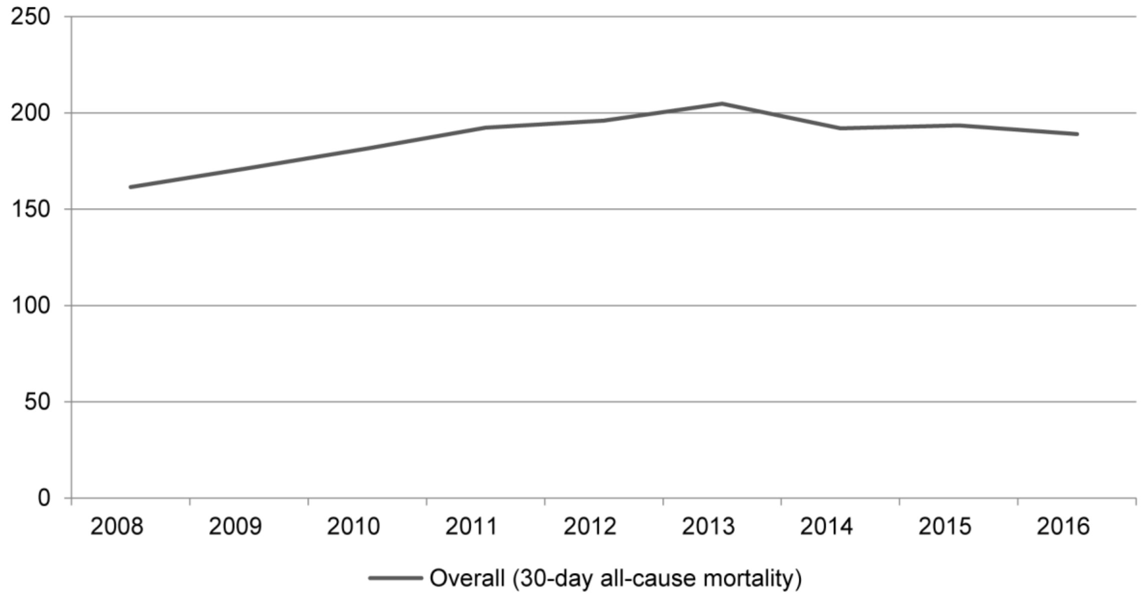

30-day all-cause mortality due to BSI increased by 17% from 162 to 189 per 100,000 hospital admissions and year during the study period (Linear regression) (p=0.03).
