# Supplementary material for: Low incidence of antibiotic-resistant bacteria in south-east Sweden: An epidemiologic study on 9268 cases of bloodstream infection
Source: PLoS One. 2020 Mar 27;15(3):e0230501. doi: 10.1371/journal.pone.0230501 (PMC7100936; doi:10.1371/journal.pone.0230501)
Supplement: S1 Table — (PDF) [file pone.0230501.s003.pdf]

S3 Table. Increase in BSI per microorganism per 100,000 hospital admissions and year, 2008-2016.

	2008	2009	2010	2011	2012	2013	2014	2015	2016	Change %*	Average yearly increase**	95% CI	p-value
Escherichia coli	277	352	443	408	470	525	574	680	534	93%	40	22.63-57.13	<0.01
Staphylococcus aureus	209	231	290	213	339	343	314	297	323	55%	14	1.72-26.05	0.03
Proteus Mirabilis	13	15	19	22	26	20	33	52	36	177%	4	1.64-5.90	<0.01
Candida albicans	16	15	30	37	23	23	45	76	59	269%	6	8.56-33.73	0.01

* Change in rate from 2008-2016 per 100,000 hospital admissions.
** Average annual increase % (linear regression per 100,000 hospital admission).
