# Supplementary material for: Low incidence of antibiotic-resistant bacteria in south-east Sweden: An epidemiologic study on 9268 cases of bloodstream infection
Source: PLoS One. 2020 Mar 27;15(3):e0230501. doi: 10.1371/journal.pone.0230501 (PMC7100936; doi:10.1371/journal.pone.0230501)
Supplement: S2 Table — (PDF) [file pone.0230501.s004.pdf]

S4 Table. Distribution of most commonly occurring microorganisms causing BSI, 2008-2016.

**Agent**	**Total (%)**	**30-day mortality**	**30-day mortality (%)**
Acinetobacter baumannii	25 (0.3)	6	24.0
Pseudomonas aeruginosa	224 (2.3)	40	17.9
Haemophilus influenzae	54 (0.6)	13	24.1
Escherichia coli	3143 (32.8)	272	8.7
Klebsiella pneumoniae	657 (6.9)	76	11.6
Klebsiella oxytoca	257 (2.7)	33	12.8
Enterobacter cloacae	279 (2.9)	30	10.8
Proteus Mirabilis	174 (1.8)	23	13.2
Enterococcus faecalis	491 (5.1)	78	15.9
Enterococcus faecium	222 (2.3)	44	19.8
Staphylococcus aureus	1885 (19.7)	356	18.9
Streptococcus pneumoniae	507 (5.3)	68	13.4
Streptococcus pyogenes (A)	162 (1.7)	17	10.5
Streptococcus agalactiae (B)	182 (1.9)	19	10.4
Streptococcus spp. (C. G)	236 (2.5)	18	7.6
Candida albicans	238 (2.5)	69	29.0
Candida spp (non albicans)	115 (1.2)	27	23.5
**Other**	**736 (7.7)**	**48**	**6.5**
