# Supplementary material for: Low incidence of antibiotic-resistant bacteria in south-east Sweden: An epidemiologic study on 9268 cases of bloodstream infection
Source: PLoS One. 2020 Mar 27;15(3):e0230501. doi: 10.1371/journal.pone.0230501 (PMC7100936; doi:10.1371/journal.pone.0230501)
Supplement: S3 Table — blood cultures. microorganism. blood culture per hospital admission. and positive blood culture per total number of blood cultures and hospital admissions). (PDF) [file pone.0230501.s005.pdf]

S5 Table. Blood culture characteristics (hospital admission. blood cultures. microorganism. blood culture per hospital admission. and positive blood culture per total number of blood cultures and hospital admissions).

Year	Hospital admission	Hospital day	Population in Östergötland	Age, median*	Total number of blood cultures	Positive blood cultures	Micro organism**	BSI	Blood cultures per hospital admission (%)***	Positive blood cultures per total number of blood cultures (%)	Positive blood cultures per hospital admission (%)
2008	69 363	378 999	423 169	69	7 860	793	712	675	11.3	10.1	1.1
2009	72 383	324 711	427 106	69	8 710	1 022	847	832	12.0	11.7	1.4
2010	72 766	337 520	429 642	69	9 211	1 100	988	969	12.7	11.9	1.5
2011	73 849	330 589	431 075	68	10 062	1 102	985	968	13.6	11.0	1.5
2012	75 516	332 719	433 784	69	11 394	1 264	1 165	1 119	15.1	11.1	1.7
2013	75 214	332 745	437 848	69	11 632	1 314	1 157	1 150	15.5	11.3	1.7
2014	75 547	344 580	442 105	69	12 041	1 316	1 235	1 199	15.9	10.9	1.7
2015	73 407	343 795	445 661	69	13 325	1 287	1 312	1 180	18.2	9.7	1.8
2016	73 033	318 081	452 105	70	14 128	1 293	1186	1 176	19.3	9.2	1.8
**Total/p-value ******					**98 363**	**10 491**	**9 587**	**9 268**	<0.01	0.11	<0.01
Change (%) *****									71%	-8.9%	64%

* Median age (25-75 percentiles) of patients from whom the blood culture were obtained
** Microorganism (bacterial and yeast isolates) typically belonging to the skin microbiota (coagulase-negative-*Staphylococci* (CoNS). *Micrococcus* spp. *Bacillus spp*. *Corynebacterium* spp. *Propionibacterium spp*.) were considered probable contaminants and excluded.
***All blood cultures taken per hospital admissions
****P-value for trend (2008-2016), Linear regression
*****Change (%) 2008 vs 2016)
