# Supplementary material for: Low incidence of antibiotic-resistant bacteria in south-east Sweden: An epidemiologic study on 9268 cases of bloodstream infection
Source: PLoS One. 2020 Mar 27;15(3):e0230501. doi: 10.1371/journal.pone.0230501 (PMC7100936; doi:10.1371/journal.pone.0230501)
Supplement: S4 Table — (PDF) [file pone.0230501.s006.pdf]

S6 Table. Incidence of BSI per microorganism and antimicrobial resistance (2008-2016).

											Regression per 100 000 hospital admissions		
	2008 R (%)	2009 R (%)	2010 R (%)	2011 R (%)	2012 R (%)	2013 R (%)	2014 R (%)	2015 R (%)	2016 R (%)	Change %	Average annual increase %	95% CI	p-value
**Gram-negative**													
**Other than Enterobacteriacae**													
Acinetobacter baumannii	**0**	**1**	**4**	**2**	**1**	**5**	**9**	**0**	**3**	**-**	**0.5**	**-0.70-1.67**	**0.36**
Tobramycin	0	0	0	100	100	20	66.7	0	33.3		4	-14.08-22.42	**0.61**
Meropenem	0	0	0	0	0	20	55.6	0	33.3		6	-1.11-12.98	**0.09**
Imipenem	0	0	0	0	0	20	44.4	0	33.3		5	-0.39-11.28	**0.06**
Ciprofloxacin	0	0	0	0	0	20	88.9	0	33.3		7	-3.74-18.56	**0.16**
Amikacin	0	0	0	0	0	20	88.9	0	0		4	-7.79-16.52	**0.42**
Pseudomonas aeruginosa	**20**	**27**	**20**	**22**	**28**	**25**	**19**	**37**	**26**	**24%**	**1**	**-1.18-3.36**	**0.30**
Ceftazidime	0	3.7	0	13.6	14.3	16	0	5.4	7.7		1	-1.86-3.58	**0.48**
Imipenem	0	3.7	5	13.6	17.9	16	15.8	5.4	7.7		1	-1.18-3.84	**0.25**
Ciprofloxacin	5	14.8	5	18.2	14.3	16	5.3	8.1	3.8		-1	-3.07-1.76	**0.54**
Tobramycin	0	0	0	9.1	0	0	0	0	0		0	-1.53-1.12	**0.73**
Meropenem	5	7.4	0	9.1	7.1	12	5.3	0	11.5		0.4	-1.53-2.24	**0.67**
Piperacillin-Tazobactam	0	7.4	0	13.6	14.3	16	15.8	13.5	19.2		3	1.25-4.56	**<0.01**
Haemophilus influenzae	**5**	**4**	**3**	**8**	**13**	**4**	**7**	**6**	**4**	**-24%**	**0**	**-1.22-1.41**	**0.87**
Penicillin G	0	0	0	0	21.4	14.3	14.3	50	25		7	2.12-11.15	**0.01**
Tetracycline	0	0	0	0	0	0	0	0	0				
Trimethoprimsulfa	0	0	0	12.5	21.4	71.4	28.6	33.3	25		7	-0.50-14.72	**0.06**
**Enterobacteriaceae**													
Escherichia coli	**192**	**255**	**322**	**301**	**355**	**395**	**434**	**499**	**390**	**93%**	**40**	**22.63-57.13**	**<0.01**
Cefotaxime	4.7	5.1	2.2	2.7	3.9	5.8	5.3	6.2	3.8		0	-0.42-0.75	**0.52**
Tobramycin	1	2	1.6	1.7	6.8	2.5	3.9	6.2	4.9		1	0.11-1.39	**0.03**
Trimethoprimsulfa	17.7	24.7	18.3	18.3	23.7	20.8	17.5	20.4	16.2		-1	-1.72-0.60	**0.29**
Ceftazidime	4.2	4.3	0.9	2.3	2.8	5.3	4.6	6.4	4.1		0	-0.33-1.00	**0.27**
Piperacillin-Tazobactam	4.2	2.4	0.9	1	3.1	2.3	0.2	4.4	2.8		0	-0.68-0.65	**0.95**

Meropenem	0	0	0	0	0	0	0	0	0				
Imipenem	0	0	0	0	0	0	0	0	0				
Ciprofloxacin	6.7	8.2	9.8	9.7	12.2	8.6	12	14.5	11		1	0.16-1.51	**0.02**
Amikacin	0	6.3	0	0	0	0	0	0	0.5		0	-1.26-0.48	**0.33**
Klebsiella pneumoniae	**48**	**38**	**85**	**74**	**92**	**69**	**70**	**92**	**89**	**76%**	**6**	**0.01-12.41**	**0.05**
Cefotaxime	0	7.9	3.5	5.4	7.6	4.3	5.7	0	2.2		0	-1.54-0.98	**0.62**
Tobramycin	0	5.3	3.5	4.1	7.6	2.9	8.6	2.2	7.9		1	-0.31-1.34	**0.18**
Trimethoprimsulfa	8.3	8.1	12.9	5.4	13	5.8	15.7	10.9	11.2		1	-0.91-1.94	**0.42**
Ceftazidime	0	7.9	3.5	2.7	8.7	4.3	7.1	0	5.6		0	-1.24-1.55	**0.80**
Piperacillin-Tazobactam	0	0	2.4	0	5.4	4.3	7.1	1.1	3.4		1	-0.26-1.63	**0.13**
Meropenem	0	0	0	0	0	0	0	0	0				
Imipenem	0	0	0	0	1.1	0	0	0	0		-	-0.16-0.16	**0.99**
Ciprofloxacin	0	2.9	4.8	4.1	6.6	2.9	12.9	2.2	1.1		0	-1.24-1.98	**0.60**
Amikacin	0	0	0	0	0	0	1.4	0	0		0	-0.13-0.26	**0.74**
Klebsiella oxytoca	**23**	**17**	**27**	**33**	**33**	**33**	**32**	**36**	**23**	**-5%**	**1**	-1.03-3.69	**0.23**
Cefotaxime	0	0	0	0	0	0	3.1	0	8.7		1	-0.09-1.95	**0.07**
Tobramycin	0	0	0	0	0	0	0	0	0				
Trimethoprimsulfa	0	5.9	0	0	3	3	6.3	0	0		0	-1.22-1.10	**0.90**
Ceftazidime	0	0	0	0	0	0	0	0	0				
Piperacillin-Tazobactam	0	0	0	3	0	3	6.3	11.1	13		2	1.18-3.26	**<0.01**
Meropenem	0	0	0	0	0	0	0	0	0				
Imipenem	0	0	0	0	0	0	0	0	0				
Ciprofloxacin	0	0	0	0	3	0	3.1	0	4.3		1	-0.09-1.15	**0.08**
Amikacin	0	0	0	0	0	0	0	0	0				
Enterobacter cloacae	**29**	**23**	**28**	**25**	**38**	**22**	**46**	**33**	**35**	**15%**	**2**	**-1.17-4.64**	**0.20**
Cefotaxime	0	17.4	14.3	24	13.2	18.2	8.7	15.2	20		1	-1.64-4.14	**0.34**
Tobramycin	0	0	0	0	0	0	0	0	0				
Trimethoprimsulfa	0	0	0	0	5.3	0	0	9.1	0		1	-0.74-1.98	**0.32**
Ceftazidime	0	8.7	14.3	24	10.5	18.2	4.3	15.2	22.9		2	-1.22-5.07	**0.19**
Piperacillin-Tazobactam	0	0	7.1	16	10.5	13.6	4.3	15.2	17.1		2	0.32-4.48	**0.03**
Meropenem	0	0	0	4	0	0	0	0	0		0	-0.67-0.49	**0.73**
Imipenem	0	0	0	0	0	0	0	0	0				
Ciprofloxacin	0	0	0	4	0	0	0	0	0		0	-0.67-0.49	**0.73**
Amikacin	0	0	0	0	0	0	0	0	0				

Proteus Mirabilis	**9**	**11**	**14**	**16**	**20**	**15**	**25**	**38**	**26**	**174%**	**4**	**1.64-5.90**	**<0.01**
Cefotaxime	0	0	0	0	0	0	0	10.5	0		1	-0.70-2.12	**0.27**
Tobramycin	0	0	0	0	0	0	4	13.2	3.8		1	-0.07-2.91	**0.06**
Trimethoprimsulfa	11.1	0	7.1	0	0	6.7	24	31.6	26.9		4	0.60-8.24	**0.03**
Ceftazidime	0	0	0	0	0	0	0	10.5	0		1	-0.70-2.13	**0.27**
Piperacillin-Tazobactam	0	0	0	0	0	0	0	0	0				
Meropenem	0	0	0	0	0	0	0	0	0				
Imipenem	0	0	0	0	0	0	0	0	0				
Ciprofloxacin	0	0	0	0	0	0	0	2.7	3.8		1	0.08-0.98	**0.03**
Amikacin	0	0	0	0	0	0	0	5.3	0		0	-0.36-1.08	**0.27**
**Gram-positive**													
**Enterococci**													
Enterococcus faecalis	**35**	**57**	**50**	**40**	**58**	**62**	**84**	**53**	**52**	**41%**	**3**	**-2.16-8.04**	**0.21**
Ampicillin	0	0	0	0	0	0	0	0	0				
Imipenem	0	0	0	0	0	1.6	0	0	0		0	-0.19-0.26	**0.73**
Linezolid	8.6	3.6	0	0	0	1.6	0	1.9	0		-1	-1.98-0.16	**0.08**
Vancomycin	0	0	0	0	1.7	0	0	1.9	0		0	-0.20-0.46	**0.38**
Enterococcus faecium	**16**	**33**	**14**	**20**	**26**	**33**	**25**	**32**	**23**	**36%**	**1**	**-1.69-4.09**	**0.36**
Ampicillin	68.8	78.8	78.6	95	73.1	97	68	84.4	87		1	-3.34-5.42	**0.59**
Imipenem	58.3	92	100	100	80.8	97	68	84.4	87		0	-6.19-6.27	**0.84**
Linezolid	0	3	0	0	0	0	0	3.1	4.3		0	-0.31-1.10	**0.23**
Vancomycin	0	0	0	0	0	0	0	0	0				
**Staphylococci**													
Staphylococcus aureus	**145**	**167**	**211**	**157**	**256**	**258**	**237**	**218**	**236**	**55%**	**14**	**1.72-26.05**	**0.03**
Cefoxitin	0	0	0	0	0	0	0.4	0	1.3		0	-0.02-0.29	**0.07**
Fusidic acid	2.8	1.2	1.9	3.8	3.1	4.7	6.3	1.8	3		0	-0.38-0.89	**0.38**
Erythromycin	4.8	2.4	4.7	3.2	4.3	3.5	3	4.6	4.7		0	-0.38-0.46	**0.84**
Clindamycin	4.1	2.4	4.3	3.2	3.1	2.3	3	3.7	4.2		0	-0.37-0.35	**0.95**
Tobramycin	0	1.8	0.9	0.6	1.2	3.5	2.5	1.8	1.3		0	-0.14-0.64	**0.18**
Linezolid	0.7	0	0	0	0	0.4	0	0	0		0	-0.16-0.05	**0.23**
Rifampicin	0	0	0	0	0	0	0.4	0	1.7		0	-0.03-0.37	**0.08**
Vancomycin		0											
**Streptococci**													

Streptococcus pneumoniae	**59**	**72**	**53**	**65**	**52**	**50**	**57**	**48**	**51**	**-18%**	**-3**	-5.67--0.32	**0.03**
Oxacillin	0	0	0	7.7	9.8	8	0	6.3	3.9		1	-0.84-2.42	**0.29**
Erythromycin	5.1	2.8	0	7.7	3.8	4	1.7	8.3	2		0	-1.17-1.28	**0.92**
Clindamycin	0	0	1.9	6.2	1.9	6	0	6.3	2		1	-0.59-1.63	**0.31**
Tetracycline	3.4	1.4	0	7.7	3.8	4	0	6.3	3.9		0	-0.85-1.41	**0.58**
Trimethoprimsulfa	5.1	5.6	1.9	7.7	9.6	14	5.2	6.3	9.8		1	-0.64-2.09	**0.25**
Penicillin G	0	0	0	3.2	2.2	4.3	0	0	2.1		0	-0.49-0.92	**0.50**
Norfloxacin	0	0	0	0	2	0	8.8	2.1	5.9		1	0.04-2.08	**0.04**
Streptococcus pyogenes (A)	**10**	**20**	**10**	**20**	**23**	**23**	**11**	**28**	**17**	**62%**	**1**	**-1.39-3.80**	**0.31**
Penicillin G	0	0	0	0	0	0	0	0	0				
Erythromycin	20	5	0	0	0	0	0	0	0		-2	-4.66-0.12	**0.06**
Clindamycin	10	5	0	0	4.3	0	0	0	0		-1	-2.49-(-)0.13	**0.03**
Penicillin G													
Streptococcus agalactiae (B)	**21**	**12**	**18**	**18**	**19**	**22**	**20**	**25**	**27**	**22%**	**1**	**0.01-2.91**	**0.05**
Penicillin G	0	0	0	0	0	0	0	0	0				
Erythromycin	4.8	8.3	5.6	11.1	0	4.5	10	0	22.2		1	-1.85-3.90	**0.43**
Clindamycin	4.8	8.3	5.6	5	0	0	5	0	14.8		0	-1.96-2.30	**0.86**
Penicillin G													
Streptococcus spp. (C. G)	**23**	**25**	**30**	**24**	**38**	**34**	**23**	**25**	**14**	**-42%**	**1**	-3.89-1.68	**0.38**
Penicillin G	0	0	0	0	0	0	0	0	0				
Erythromycin	4.3	0	0	0	0	0	0	0	14.3		1	-1.11-2.89	**0.33**
Clindamycin	0	4	0	0	0	0	0	0	14.3		1	-0.90-2.96	**0.25**

Yeast

Candida albicans	**11**	**11**	**22**	**27**	**17**	**17**	**34**	**56**	**43**		**6**	**2.24-10.12**	**0.01**
Fluconazole	0	0	0	0	0	0	0	0	0				
Flucytosin	0	0	0	0	0	0	0	3.6	4.7		1	0.11-1.24	**0.03**
Amphotericin	0	0	0	0	0	0	0	0	0				
Anidulafungin	0	0	0	0	0	0	0	0	0				
Voriconazole	0	0	0	0	0	0	0	0	0				
Candida spp (non albicans)	**5**	**9**	**11**	**17**	**22**	**11**	**21**	**9**	**10**		**1**	**1.68-3.10**	**0.51**
Fluconazole	0	0	0	5.9	13.6	9.1	9.5	33.3	20		5	1.88-7.28	**0.01**
Flucytosin	0	0	0	0	0	0	9.5	22.2	10		3%	0.48-5.21	**0.02**

Amphotericin	0	0	0	0	0	0	0	0	10	1%	-0.33-2.16	**0.13**
Anidulafungin	0	0	0	0	0	0	0	0	0			
Voriconazole	0	0	9.1	0	0	0	0	0	10	0%	-1.34-2.33	**0.54**
