# Supplementary material for: Low incidence of antibiotic-resistant bacteria in south-east Sweden: An epidemiologic study on 9268 cases of bloodstream infection
Source: PLoS One. 2020 Mar 27;15(3):e0230501. doi: 10.1371/journal.pone.0230501 (PMC7100936; doi:10.1371/journal.pone.0230501)
Supplement: S5 Table — (PDF) [file pone.0230501.s007.pdf]

S7 Table. Antibacterials for systemic use (J01) excluding metenamine (J01-J01XX05) measured as defined-daily-doses (DDD) per 1.000 inhabitants and day (TIND).

	2008	2009	2010	2011	2012	2013	2014	2015	2016	Change %*	95% CI**	p-value**
Used on hospital wards and polyclinics measured as DDD/TIND	1.28	1.25	1.31	1.42	1.45	1.48	1.50	1.50	1.50	17	0.02-0.05	<0.01
Dispensed to outpatients measured as DDD/TIND	11.75	11.47	11.61	11.36	11.26	10.84	10.53	10.28	10.22	-13	-0.25-(-)0.16	<0.01

* Change in rate from 2008-2016
**Linear regression, DDD/TIND, 2008-2016.
