# Supplementary material for: Low incidence of antibiotic-resistant bacteria in south-east Sweden: An epidemiologic study on 9268 cases of bloodstream infection
Source: PLoS One. 2020 Mar 27;15(3):e0230501. doi: 10.1371/journal.pone.0230501 (PMC7100936; doi:10.1371/journal.pone.0230501)
Supplement: S6 Table — (PDF) [file pone.0230501.s008.pdf]

S8 Table. Amount of antibacterials for systemic use (J01) used on hospital wards and polyclinics measured in defined-daily-doses (DDD) per 1,000 hospital days.

	2008	2009	2010	2011	2012	2013	2014	2015	2016	Change%*	95% CI**	p-value**
Tetracyclines (J01A)	87.6	87.1	80.3	96.2	102	82.6	85.9	82.0	75.0	-14	-3.62-1.35	0.32
Penicillins with extended spectrum (J01CA)	50.3	64.2	60.2	61.3	59.3	68.6	69.2	70.1	74.4	48	1.16-3.48	<0.01
Beta-lactamase sensitive penicillins (J01CE)	41.1	45.7	43.9	54.9	59.9	62.2	62.5	65.9	73.5	79	3.06-4.76	<0.01
Beta-lactamase resistant penicillins (J01CF)	65.8	90.8	90.8	106.1	122.9	130.1	126.0	119.6	137.8	109	4.69-10.94	<0.01
Combination of penicillins (J01CR)	26.4	31.3	41.1	50.9	46.2	50.4	55.7	64.8	85.5	224	4.15-8.04	<0.01
Cephalosporins (J01DB-DE)	81.2	93.9	90.9	89.8	94.4	98	94.6	95.8	100.9	24	0.59-2.74	0.01
Carbapenems (J01DH)	31.3	36.1	42	46.4	47	48.6	46.7	49.9	48.0	53	0.95-3.04	<0.01
Sulfonamides and trimethoprim J01E)	25.4	22.8	20.1	18.7	17.6	15.7	17.3	17.4	20.0	-21	-1.49-(-)0.06	0.04
Macrolides. lincosamides and streptogramins (J01F)	23.5	27.2	30.5	32.6	32.7	35.3	31.1	31.6	35.1	49	0.29-1.83	0.01
Aminoglycosides (J01GB)	6.2	6.2	9.7	11.5	11	9.8	10.2	9.3	7.8	26	-0.34-0.84	0.35
Fluoroquinolones (J01MA)	56.4	62.9	66.4	70.5	63.5	63.6	59.7	63.5	68.8	22	-0.81-1.85	0.39
Vancomycin (J01XA01)	7.0	8.4	9.9	10.3	9.6	13	11.6	12.9	15.6	123	0.60-1.20	<0.01
Other	21.7	22.2	23.2	25.8	26.8	31.3	31.2	28.7	34.2	58	1.01-2.02	<0.01
**Total amount of antibacterials for systemic use (J01)**	**524.0**	**598.9**	**609.3**	**675.1**	**692.9**	**709.2**	**701.8**	**711.6**	**776.5**	**48**	**17.81-34.43**	**<0.01**

* Change in rate from 2008-2016
**Linear regression, DDD/TIND, 2008-2016.
