# Supplementary material for: Low incidence of antibiotic-resistant bacteria in south-east Sweden: An epidemiologic study on 9268 cases of bloodstream infection
Source: PLoS One. 2020 Mar 27;15(3):e0230501. doi: 10.1371/journal.pone.0230501 (PMC7100936; doi:10.1371/journal.pone.0230501)
Supplement: S7 Table — (PDF) [file pone.0230501.s009.pdf]

S9 Table. Amount of antibacterials for systemic use (J01) used on hospital wards and polyclinics measured in defined-daily doses (DDD) per hospital admission.

	2008	2009	2010	2011	2012	2013	2014	2015	2016	Change%*	95% CI**	p-value**
Tetracyclines (J01A)	0.48	0.39	0.37	0.43	0.45	0.37	0.39	0.38	0.33	-31	-0.02-0.00	0.06
Penicillins with extended spectrum (J01CA)	0.27	0.29	0.28	0.27	0.26	0.30	0.32	0.33	0.32	19	0.00-0.01	0.02
Beta-lactamase sensitive penicillins (J01CE)	0.22	0.21	0.20	0.25	0.26	0.27	0.29	0.31	0.32	46	0.01-0.02	<0.01
Beta-lactamase resistant penicillins (J01CF)	0.36	0.41	0.42	0.47	0.54	0.58	0.57	0.56	0.60	67	0.02-0.04	<0.01
Combination of penicillins (J01CR)	0.14	0.14	0.19	0.23	0.20	0.22	0.25	0.30	0.37	164	0.02-0.03	<0.01
Cephalosporins (J01DB-DE)	0.44	0.42	0.42	0.40	0.42	0.43	0.43	0.45	0.44	0	-0.00-0.01	0.25
Carbapenems (J01DH)	0.17	0.16	0.20	0.21	0.21	0.21	0.21	0.23	0.21	24	0.00-0.01	0.01
Sulfonamides and trimethoprim J01E)	0.14	0.10	0.09	0.08	0.08	0.07	0.08	0.08	0.09	-36	-0.01-0.00	0.06
Macrolides. lincosamides and streptogramins (J01F)	0.13	0.12	0.14	0.15	0.14	0.16	0.14	0.15	0.15	15	0.00-0.01	0.04
Aminoglycosides (J01GB)	0.03	0.03	0.04	0.05	0.05	0.04	0.05	0.04	0.03	0	-0.00-0.00	0.59
Fluoroquinolones (J01MA)	0.31	0.28	0.31	0.32	0.28	0.28	0.27	0.30	0.30	3	-0.01-0.00	0.50
Vancomycin (J01XA01)	0.04	0.04	0.05	0.05	0.04	0.06	0.05	0.06	0.07	75	0.00-0.01	0.01
Other	0.12	0.10	0.11	0.12	0.12	0.14	0.14	0.13	0.15	25	0.00-0.01	0.01
**Total amount of antibacterials for systemic use (J01)**	**2.86**	**2.69**	**2.83**	**3.02**	**3.05**	**3.14**	**3.20**	**3.33**	**3.38**	**18**	**0.06-0.10**	**0.01**

* Change in rate from 2008-2016
**Linear regression, DDD/TIND, 2008-2016.
