# Supplementary material for: Low incidence of antibiotic-resistant bacteria in south-east Sweden: An epidemiologic study on 9268 cases of bloodstream infection
Source: PLoS One. 2020 Mar 27;15(3):e0230501. doi: 10.1371/journal.pone.0230501 (PMC7100936; doi:10.1371/journal.pone.0230501)
Supplement: S8 Table — (PDF) [file pone.0230501.s010.pdf]

S10 Table. Categories and agents used to define MDR (worksheet for categorizing isolates).

**Gram-negative**	
**Other than Enterobacteriacae**	
Acinetobacter baumannii	
Aminoglycosides	Tobramycin
Carbapenems	Meropenem
Carbapenems	Imipenem
Fluoroqinolones	Ciprofloxacin
Aminoglycosides	Amikacin
Pseudomonas aeruginosa	
Extended-spectrum cephalosporins; 3rd and 4th generation cephalosporins	Ceftazidime
Carbapenems	Imipenem
Fluoroqinolones	Ciprofloxacin
Aminoglycosides	Tobramycin
Carbapenems	Meropenem
Antipseudomonal penicillins +b-lactamase inhibitors	Piperacillin-Tazobactam
Haemophilus influenzae	
Penicillins	Penicillin G
Tetracyclines	Tetracycline
Folate pathway inhibitors	Trimethoprimsulfa
**Enterobacteriaceae**	
Escherichia coli	
Extended-spectrum cephalosporins; 3rd and 4th generation cephalosporins	Cefotaxime
Aminoglycosides	Tobramycin
Folate pathway inhibitors	Trimethoprimsulfa
Extended-spectrum cephalosporins; 3rd and 4th generation cephalosporins	Ceftazidime
Antipseudomonal penicillins +b-lactamase inhibitors	Piperacillin-Tazobactam
Carbapenems	Meropenem
Carbapenems	Imipenem

Fluoroqinolones	Ciprofloxacin
Aminoglycosides	Amikacin
Klebsiella pneumoniae	
Extended-spectrum cephalosporins; 3rd and 4th generation cephalosporins	Cefotaxime
Aminoglycosides	Tobramycin
Folate pathway inhibitors	Trimethoprimsulfa
Extended-spectrum cephalosporins; 3rd and 4th generation cephalosporins	Ceftazidime
Antipseudomonal penicillins +b-lactamase inhibitors	Piperacillin-Tazobactam
Carbapenems	Meropenem
Carbapenems	Imipenem
Fluoroqinolones	Ciprofloxacin
Aminoglycosides	Amikacin
Klebsiella oxytoca	
Extended-spectrum cephalosporins; 3rd and 4th generation cephalosporins	Cefotaxime
Aminoglycosides	Tobramycin
Folate pathway inhibitors	Trimethoprimsulfa
Extended-spectrum cephalosporins; 3rd and 4th generation cephalosporins	Ceftazidime
Antipseudomonal penicillins +b-lactamase inhibitors	Piperacillin-Tazobactam
Carbapenems	Meropenem
Carbapenems	Imipenem
Fluoroqinolones	Ciprofloxacin
Aminoglycosides	Amikacin
**Enterobacter cloacae**	
Extended-spectrum cephalosporins; 3rd and 4th generation cephalosporins	Cefotaxime
Aminoglycosides	Tobramycin
Folate pathway inhibitors	Trimethoprimsulfa
Extended-spectrum cephalosporins; 3rd and 4th generation cephalosporins	Ceftazidim

Antipseudomonal penicillins +b-lactamase inhibitors	Piperacillin-Tazobactam
Carbapenems	Meropenem
Carbapenems	Imipenem
Fluoroqinolones	Ciprofloxacin
Aminoglycosides	Amikacin
Proteus Mirabilis	
Extended-spectrum cephalosporins; 3rd and 4th generation cephalosporins	Cefotaxime
Aminoglycosides	Tobramycin
Folate pathway inhibitors	Trimethoprimsulfa
Extended-spectrum cephalosporins; 3rd and 4th generation cephalosporins	Ceftazidime
Antipseudomonal penicillins +b-lactamase inhibitors	Piperacillin-Tazobactam
Carbapenems	Meropenem
Carbapenems	Imipenem
Fluoroqinolones	Ciprofloxacin
Aminoglycosides	Amikacin
**Gram-positive**	
**Enterococci**	
Enterococcus faecalis	
Penicillins	Ampicillin
Carbapenems	Imipenem
Oxazolidinones	Linezolid
Glycopeptides	Vancomycin
Enterococcus faecium	
Penicillins	Ampicillin
Carbapenems	Imipenem
Oxazolidinones	Linezolid
Glycopeptides	Vancomycin
**Staphylococci**	
Staphylococcus aureus	

Anti-staphylococcal b-lactams (or cephamycins)	Cefoxitin
Fucidanes	Fusidic acid
Macrolides	Erythromycin
Lincosamides	Klindamycin
Aminoglycosides	Tobramycin
Oxazolidinones	Linezolid
Ansamycins	Rifampicin
Glycopeptides	Vancomycin
**Streptococci**	
Streptococcus pneumoniae	
Anti-staphylococcal b-lactams (or cephamycins)	Oxacillin
Macrolides	Erythromycin
Lincosamides	Clindamycin
Tetracyclines	Tetracycline
Folate pathway inhibitors	Trimethoprimsulfa
Penicillins	Penicillin G
Fluoroquinolones	Norfloxacin
Streptococcus pyogenes (A)	
Penicillins	Penicillin G
Macrolides	Erythromycin
Lincosamides	Klindamycin
	Penicillin G
Streptococcus agalactiae (B)	
Penicillins	Penicillin G
Macrolides	Erythromycin
Lincosamides	Clindamycin
	Penicillin G
Streptococcus spp. (C. G)	
Penicillins	Penicillin G
Macrolides	Erythromycin
Lincosamides	Clindamycin
