# Supplementary material for: Low incidence of antibiotic-resistant bacteria in south-east Sweden: An epidemiologic study on 9268 cases of bloodstream infection
Source: PLoS One. 2020 Mar 27;15(3):e0230501. doi: 10.1371/journal.pone.0230501 (PMC7100936; doi:10.1371/journal.pone.0230501)
Supplement: S9 Table — (PDF) [file pone.0230501.s011.pdf]

**S11 Table. Comorbidity per 100,000 hospital admissions and year (overall BSIs and 30-day all-cause mortality).**

| Overall BSIs (n = 9,268) | 2008 | 2009 | 2010 | 2011 | 2012 | 2013 | 2014 | 2015 | 2016 | Change (%) | Average annual increase | 95% CI | p-value |
|---|---|---|---|---|---|---|---|---|---|---|---|---|---|
| Number of comorbidities | | | | | | | | | | | | | |
| **0** | 248 | 344 | 352 | 382 | 408 | 408 | 458 | 489 | 435 | 75% | 24 | 14-34 | <0.01 |
| **1** | 268 | 334 | 383 | 340 | 387 | 403 | 384 | 381 | 457 | 71% | 16 | 7-25 | <0.01 |
| **2** | 185 | 191 | 219 | 219 | 249 | 294 | 271 | 247 | 233 | 26% | 9 | 0.6-17 | 0.04 |
| **>2** | 272 | 280 | 378 | 370 | 438 | 424 | 474 | 490 | 485 | 78% | 29 | 20-37 | <0.01 |
| | | | | | | | | | | | | | |
| **30-day mortality (n = 1,237)** | | | | | | | | | | | | | |
| Number of comorbidities | | | | | | | | | | | | | |
| **0** | 23 | 18 | 19 | 24 | 24 | 25 | 17 | 25 | 25 | 9% | 0.4 | -0.6-1.4 | 0.39 |
| **1** | 40 | 52 | 45 | 57 | 50 | 56 | 46 | 37 | 33 | -18% | -1.3 | -3.8-1.2 | 0.27 |
| **2** | 48 | 47 | 55 | 42 | 50 | 49 | 46 | 41 | 33 | -31% | -1.5 | -3.1-0.2 | 0.07 |
| **>2** | 50 | 54 | 62 | 69 | 72 | 74 | 82 | 91 | 99 | 98% | 6 | 5.1-6.6 | <0.01 |
