# Supplementary material for: Low incidence of antibiotic-resistant bacteria in south-east Sweden: An epidemiologic study on 9268 cases of bloodstream infection
Source: PLoS One. 2020 Mar 27;15(3):e0230501. doi: 10.1371/journal.pone.0230501 (PMC7100936; doi:10.1371/journal.pone.0230501)
Supplement: S10 Table — (PDF) [file pone.0230501.s012.pdf]

S12 Table. Antibiotic resistance in Enterobacteriaceae (2008-2016)

										Regression per 100.000 hospital admissions		
	**2008**	**2009**	**2010**	**2011**	**2012**	**2013**	**2014**	**2015**	**2016**	**AAI %***	**95% CI**	**p-value**
Enterobacteriaceae (n)	355	396	525	496	581	582	645	723	600	49	(27.42-69.89)	<0.01
**Resistance**												
Fluoroquinolone (%)	3.66	5.56	6.86	6.65	8.61	6.19	9.61	10.37	7.67	0.8	(0.21-1.32)	0.01
PTZ (%)	2.27	1.52	1.33	1.61	3.44	2.75	1.55	4.43	3.83	0.4	(-0.03-0.75)	0.07
TMP/SMX (%)	10.98	16.94	13.50	11.91	17.06	15.15	14.72	17.54	13.36	0.3	(-0.65-1.22)	0.49
Cephalosporin (%)	2.54	5.05	2.67	3.63	4.65	5.15	5.12	5.67	5.17	0.4	(0.06-0.75)	0.03

*AAI = Averageg annual increase %
