# Supplementary material for: Low incidence of antibiotic-resistant bacteria in south-east Sweden: An epidemiologic study on 9268 cases of bloodstream infection
Source: PLoS One. 2020 Mar 27;15(3):e0230501. doi: 10.1371/journal.pone.0230501 (PMC7100936; doi:10.1371/journal.pone.0230501)
Supplement: S11 Table — (PDF) [file pone.0230501.s013.pdf]

S13 Table. The Charlson Comorbidity Index (Updated Weight)

Comorbid Condition	ICD-9-CM Diagnosis Codes	ICD-10-CA Diagnosis Codes	Weight	Updated Weight
Myocardial Infarction	410, 412	I21, I22, I25.2	1	0
Congestive Heart Failure	398.91, 402.01, 402.11, 402.91, 404.01, 404.03, 404.11, 404.13, 404.91, 404.93, 425.4–425.9, 428 (hosp), 398, 402, 425, 428 (med)	I09.9, I11.0, I13.0, I13.2, I25.5, I42.0, I42.5–I42.9,I43, I50, P29.0	1	2
Peripheral Vascular disease	093.0, 437.3, 440, 441, 443.1–443.9, 447.1, 557.1, 557.9, V43.3 (hosp)<br>440, 441, 443, 447, 557 (med)	I70, I71, I73.1, I73.8, I73.9, I77.1, I79.0, I79.2, K55.1, K55.8, K55.9, Z95.8, Z95.9	1	0
Cerebrovascular Disease	362.34, 430–438 (hosp)<br>430–438 (med)	G45, G46, H34.0, I60–I69	1	0
Dementia	290, 294.1, 331.2 (hosp)<br>290, 294, 331 (med)	F00–F03, F05.1, G30, G31.1	1	2
Chronic Pulmonary Disease	416.8, 416.9, 490–505, 506.4, 508.1, 508.8 (hosp)<br>416, 490–496, 500–505 (med)	I27.8, I27.9,J40–J47, J60–J67 J68.4, J70.1, J70.3	1	1
Connective Tissue Disease-Rheumatic Disease	446.5, 710.0–710.4, 714.0–714.2, 714.8, 725 (hosp)<br>446, 710, 714, 725 (med)	M05, M06, M31.5, M32–M34, M35.1, M35.3, M36.0	1	1
Peptic Ulcer Disease	531–534	K25–K28	1	0
Mild Liver Disease	070.22, 070.23, 070.32, 070.33, 070.44, 070.54, 070.6, 070.9, 570, 571, 573.3, 573.4, 573.8, 573.9, V42.7 (hosp)<br>070, 570, 571, 573 (med)	B18, K70.0–K70.3, K70.9, K71.3–K71.5, K71.7, K73, K74, K76.0, K76.2–K76.4, K76.8, K76.9, Z94.4	1	2
Diabetes without Chronic	250.0–250.3, 250.8, 250.9 (hosp)	E10.0, E10.1, E10.6, E10.8, E10.9, E11.0,	1	0

Complications	250 (med)	E11.1, E11.6, E11.8, E11.9, E12.0, E12.1, E12.6, E12.8, E12.9, E13.0, E13.1, E13.6, E13.8, E13.9, E14.0, E14.1, E14.6, E14.8, E14.9		
Diabetes with Chronic Complications	250.4–250.7 (med n/a)	E10.2–E10.5, E10.7, E11.2–E11.5, E11.7, E12.2–E12.5, E12.7, E13.2–E13.5, E13.7, E14.2–E14.5, E14.7	2	1
Paraplegia and Hemiplegia	334.1, 342, 343, 344.0–344.6, 344.9 (hosp), 334, 342–344 (med)	G04.1, G11.4, G80.1, G80.2, G81, G82, G83.0–G83.4, G83.9	2	2
Renal Disease	403.01, 403.11, 403.91, 404.02, 404.03, 404.12, 404.13, 404.92, 404.93, 582, 583.0–583.7, 585, 586, 588.0. V42.0, V45.1, V56 (hosp), 403, 582, 583, 585, 586, 588, V56 (med)	I12.0, I13.1, N03.2–N03.7, N052–N05.7, N18, N19, N25.0, Z49.0–Z49.2, Z94.0, Z99.2	2	1
Cancer *	140–172, 174–195.8, 200–208, 238.6 (hosp), 140–172, 174–195, 200–208, 238 (med)	C00–C26, C30–C34, C37–C41, C43, C45–C58, C60–C76, C81–C85, C88, C90–C97	2	2
Moderate or Severe Liver Disease	456.0–456.2, 572.2–572.4, 572.8 (hosp), 456, 572 (med)	I85.0, I85.9, I86.4, I98.2, K70.4, K71.1, K72.1, K72.9, K76.5–K76.7,	3	4
Metastatic Carcinoma**	196–199	C77–C80	6	6
HIV/AIDS	042–044	B20–B22, B24	6	4

Abbreviations: AIDS, acquired immunodeficiency syndrome; HIV, human immunodeficiency virus. * Including lymphoma and leukemia, except malignant neoplasm of skin. ** or Metastatic solid tumor.
