# Supplementary material for: Low incidence of antibiotic-resistant bacteria in south-east Sweden: An epidemiologic study on 9268 cases of bloodstream infection
Source: PLoS One. 2020 Mar 27;15(3):e0230501. doi: 10.1371/journal.pone.0230501 (PMC7100936; doi:10.1371/journal.pone.0230501)
Supplement: Attachment — Submitted filename: Response to Reviewers.docx [file pone.0230501.s014.docx]

Paper PONE-D-19-29217

To the Editor

Thank you for reviewing our manuscript *“Low incidence of antibiotic-resistant bacteria in south-east Sweden: an epidemiologic study on 9268 cases of bloodstream infection”* and for inviting us to submit a revised version of the manuscript that addresses the points raised in the review process.

We have revised the text according to the referees’ suggestions and below are our responses to the referees’ questions and comments.

We have also uploaded a 'Revised Manuscript with Track Changes'.

Please let us know if there is anything in our reply that needs further clarification.

We hope our revision is now acceptable for publication.

Yours faithfully

......................................................................................

Håkan Hanberger, MD, Professor

Div. Infectious Diseases, Faculty of Medicine, Linköping University, Sweden Phone +46705797102, Skype hakan.hanberger
Low incidence of antibiotic-resistant bacteria in south-east Sweden: an epidemiologic study on 9268 cases of bloodstream infection.
PLOS ONE

**Response to reviewers**

**Editor:** The reviewers presented several comments, see below. Please address their comments and revise accordingly. The reviewers were not in agreement concerning the manuscript, with the main concern raised being missing variables important for the analysis. I am aware that these variables would not be possible to complete from your database, however this issue should be clearly stated in the limitations section.

**AU:** Thank you, we have made additions to the limitations of the study – see Lines 399-402.

- The scope of this paper was not to investigate appropriate antibiotic treatment, site of infection or severity of illness since this information was not available in the database. However, this is now included as a limitation of the study.

**Editor:** We would appreciate receiving your revised manuscript by 22-Jan-2020. When you are ready to submit your revision, log on to https://www.editorialmanager.com/pone/ and select the 'Submissions Needing Revision' folder to locate your manuscript file.

If you would like to make changes to your financial disclosure, please include your updated statement in your cover letter.

**AU:** There are no financial disclosures to be made.

**Editor Sent:** den 17 januari 2020 21:43: Dear Prof Hanberger, Thank you for your recent message. We received your request to extend the resubmission date for your revised manuscript. It is perfectly fine to have an extension in order to complete your revisions. I **have extended your due date to 29 February 2020.** Please do take the time to make the desired changes, and let us know if you will not be submitting within six months of your decision date. Please feel free to contact us if you have any further questions or concerns. Kind regards, Jessica Measham Staff EO PLOS ONE

**AU:** Thank you.

**Editor:** To enhance the reproducibility of your results, we recommend that if applicable you deposit your laboratory protocols in protocols.io, where a protocol can be assigned its own identifier (DOI) such that it can be cited independently in the future. For instructions see: [http://journals.plos.org/plosone/s/submission-guidelines#loc-laboratory-protocols](http://journals.plos.org/plosone/s/submission-guidelines#loc-laboratory-protocols)

**AU:** This has been performed and the schematic outline of the study has been added (see Fig1. Flow-chart. Study design below).

**Editor:** Please include the following items when submitting your revised manuscript:

- A rebuttal letter that responds to each point raised by the academic editor and reviewer(s). This letter should be uploaded as separate file and labeled **'Response to Reviewers'.**
- A marked-up copy of your manuscript that highlights changes made to the original version. This file should be uploaded as separate file and labeled **'Revised Manuscript with Track Changes'.**
- An unmarked version of your revised paper without tracked changes. This file should be uploaded as separate file and labeled **'Manuscript'.**

**AU:** This has been done.

**Editor:** Please note while forming your response, if your article is accepted, you may have the opportunity to make the peer review history publicly available. The record will include editor decision letters (with reviews) and your responses to reviewer comments. If eligible, we will contact you to opt in or out. We look forward to receiving your revised manuscript. Kind regards, Dafna Yahav Academic Editor PLOS ONE
Journal Requirements: When submitting your revision, we need you to address these additional requirements.

1. Please ensure that your manuscript meets PLOS ONE's style requirements, including those for file naming. The PLOS ONE style templates can be found at http://www.journals.plos.org/plosone/s/file?id=wjVg/PLOSOne_formatting_sample_main_body.pdf and http://www.journals.plos.org/plosone/s/file?id=ba62/PLOSOne_formatting_sample_title_authors_affiliations.pdf

2. We ask that you please include in your methods section, a statement that the need for informed consent was waived by your ethics committee. Thank you for including this information in your on-line ethics statement.

**AU:** This has been included. The local Ethics Committee decided that informed consent was not necessary. No details of the patients are disclosed and thus patient confidentiality is secure. This has been included in the manuscript Line 213.

3. PLOS requires an ORCID iD for the corresponding author in Editorial Manager on papers submitted after December 6th, 2016. Please ensure that you have an ORCID iD and that it is validated in Editorial Manager. To do this, go to ‘Update my Information’ (in the upper left-hand corner of the main menu), and click on the Fetch/Validate link next to the ORCID field. This will take you to the ORCID site and allow you to create a new iD or authenticate a pre-existing iD in Editorial Manager. Please see the following video for instructions on linking an ORCID iD to your Editorial Manager account: <https://www.youtube.com/watch?v=_xcclfuvtxQ>

**AU:** This has been done.

4. We note you have included a table to which you do not refer in the text of your manuscript. Please ensure that you refer to Table 1 in your text; if accepted, production will need this reference to link the reader to the Table.

**AU:** This has been done: Line 285.


[Note: HTML markup is below. Please do not edit.]

Reviewers' comments:

Reviewer's Responses to Questions

**Comments to the Author**

1. Is the manuscript technically sound, and do the data support the conclusions?

The manuscript must describe a technically sound piece of scientific research with data that supports the conclusions. Experiments must have been conducted rigorously, with appropriate controls, replication, and sample sizes. The conclusions must be drawn appropriately based on the data presented.

Reviewer #1: Partly

Reviewer #2: Partly

Reviewer #3: Yes

Reviewer #4: Partly

Reviewer #5: Partly

**AU:** We have tried to improve the text and illustrations in order that the data are more in line with the conclusions.

2. Has the statistical analysis been performed appropriately and rigorously?

Reviewer #1: Yes

Reviewer #2: I Don't Know

Reviewer #3: Yes

Reviewer #4: Yes

Reviewer #5: Yes

**AU:** Our professional statistician has once more checked that analyses are correct.

3. Have the authors made all data underlying the findings in their manuscript fully available?

The [PLOS Data policy](http://www.plosone.org/static/policies.action#sharing) requires authors to make all data underlying the findings described in their manuscript fully available without restriction, with rare exception (please refer to the Data Availability Statement in the manuscript PDF file). The data should be provided as part of the manuscript or its supporting information, or deposited to a public repository. For example, in addition to summary statistics, the data points behind means, medians and variance measures should be available. If there are restrictions on publicly sharing data—e.g. participant privacy or use of data from a third party—those must be specified.

Reviewer #1: Yes

Reviewer #2: No

Reviewer #3: Yes

Reviewer #4: Yes

Reviewer #5: Yes

**AU:** We have made sure that our laboratory protocols are available.

4. Is the manuscript presented in an intelligible fashion and written in standard English?

PLOS ONE does not copyedit accepted manuscripts, so the language in submitted articles must be clear, correct, and unambiguous. Any typographical or grammatical errors should be corrected at revision, so please note any specific errors here.

Reviewer #1: Yes

Reviewer #2: Yes

Reviewer #3: Yes

Reviewer #4: Yes

Reviewer #5: Yes

**AU:** Thank you.

5. Review Comments to the Author

Please use the space provided to explain your answers to the questions above. You may also include additional comments for the author, including concerns about dual publication, research ethics, or publication ethics. (Please upload your review as an attachment if it exceeds 20,000 characters)

**Reviewer #1: General comment**
This is a retrospective cohort study analysing blood culture isolates form a Swedish county, covering the years 2008-2016. The authors demonstrate that BSI have been increasing over time, that BSI caused by resistant pathogens are rare in this specific setting and that 30-day mortality is mainly related to the patient comorbidities.

1. The main limitation is the lack of important confounder variables in the mortality analysis (e.g. appropriate antibiotic treatment, site of infection, severity of illness).

**AU:** The scope of this paper was not to investigate appropriate antibiotic treatment, site of infection or severity of illness since this information was not available in the database. However, we accept this as being a limitation of the study. We have included this as a main limitation of the study. The text now reads: **(see Line 399)**

“A major limitation is that individual patient data on severity of disease, site of infection and appropriate antibiotic treatment were not available in our database.”

2. The discussion needs restructuring.

**AU:** We agree and have improved the structure of the discussion according to the comments of referee/reviewers 1-5.

Detailed comments
Introduction
**3.** - line 78: Write out BSI when first mentioning
**AU:** This has been done. The text now reads: **(see Line 78)**

“The high burden of bloodstream infection (BSI) and increasing prevalence of BSI caused by antimicrobial-resistant (AMR) and multidrug-resistant (MDR) bacteria is a serious threat to global public health [1-6].”

Methods
**4.** - line 118: are isolates from outpatients outside the four hospitals also represented in the database?

**AU:** This has been done. The text now reads: **(see Line 143)**

“All isolated microorganisms from outpatient care and hospital admissions were analyzed at the species level.”

**5.** - line 195: you are performing dozens of statistical tests in your analysis. Consider adjusting the p-values for multiple testing, or mention this as a limitation.

**AU:** We have now mentioned this under Limitations. The text now reads: **(see Line 405)**

“Since this was mainly an explorative analysis, we have not made adjustments to the p-values; a measure usually taken when testing multiple hypotheses.”

Results
**6.** - line 219: 9268 BSI from how many patients?

**AU:** 8,498 patients were included. Data were reported as BSI episodes according to the definitions “positive blood culture”, “BSI episode”, and “new BSI episode” provided in the text. We have now added a flow-chart to the Methods section. **See Line 137.**

**7.** - line 231: regarding increased 30d mortality over time: Could it be that follow-up was different over time? In other words, how sure can you be about those patients for whom no death was reported? Please comment.

**AU:** Sweden has a national death register where date, time and place of death are recorded. We extracted data and matched these with our clinical data. This procedure did not change during the period of this study.

**8.** - line 254: what is the difference between the 17% increase mentioned on line 253 and the 48% on line 254?

**AU:** 17% = DDD/TIND (1,000 inhabitants and day), 48% = DDD/1,000 hospital days. In previous studies, both DDD/TIND and DDD/1,000 hospital days have been presented. Both were included to show differences due to the denominator.

**9.** - line 256: what does “analyzed at the 5th ATC level” mean?

**AU:** In the ATC classification system, active substances are classified in a five-level hierarchy. The 5th ATC level is the basic chemical compound.

**10.** - Table 1: Comment: From an epidemiological perspective, separation between MDR and non-MDR ESBL does not make much sense.

**AU:** We understand it may be a problem from an epidemiologic perspective, but in the design of this study we decided to use the generally accepted definition of multidrug resistance (MDR) *i.e.* non-susceptibility to at least 1 agent in ≥3 antimicrobial classes (Falagas, J Med Microbiol, 2006 and Magiorakos, A.P., et al., Clin Microbiol Infect, used by WHO, ECDC).

However, application of this MDR definition to our data excludes a significant number of ESBL-producing Enterobacteriaceae not fulfilling the MDR criteria thereby underestimating the frequency of AMR. We thus report not only MDR but also AMR without multidrug resistance (MRSA, ESBL-producing Enterobacteriaceae, vancomycin-resistant enterococci not fulfilling the MDR criteria). As seen in Table 3, 76% of AMR-BSIs were caused by MDR-BSIs and 24% were AMR-BSIs without multidrug resistance including ESBL, MRSA, and VRE. Presentation of data in this paper enables comparison with ECDC data on the frequency of 3rd generation Cephalosporin-resistant Enterobacteriaceae (ESBL phenotype), MRSA, VRE and MDR E. coli/Klebsiella.

AMR and MDR definitions are included in Methods (see Lines 169-173 in Methods section) and we have added a comment in Discussion. The text now reads: **(see Line 381)**

“In the design of this study we decided to use the generally accepted definition of multidrug resistance (MDR) *i.e.* non-susceptibility to at least 1 agent in ≥3 antimicrobial classes (Falagas, J Med Microbiol, 2006 and Magiorakos, A.P., et al., Clin Microbiol Infect)

However, application of this MDR definition to our data would exclude a significant number of MRSA, ESBL-producing Enterobacteriaceae not fulfilling the MDR criteria thereby underestimating the frequency of AMR. Thus we report MDR but also AMR without multidrug resistance (MRSA, ESBL-producing Enterobacteriaceae, vancomycin-resistant enterococci not fulfilling the MDR criteria)”.

**11.** - Table 3: p-value for gender is missing

**AU:** The text now reads: **(see Line 314)**

“P-value = 0.19 (Chi^2^-analysis)”

**12.** - Table 4: a limitation of your analysis is the small number of potential confounders. What about severity of disease? Site of infection? Lower mortality with AMR is strange and is probably a result of residual confounding.

**AU:** We agree that this is a limitation and have included it as such. The text now reads: **(see Line 399)**

“A major limitation is that individual patient data on severity of disease, site of infection and appropriate antibiotic treatment were not available in our database.”

Discussion
**13.** - Paragraph 1 is not well structured. You are intermingling all aspects of your results in the first paragraph, which is puzzling to the reader. This should be a summary of your main findings. In general, do not repeat details of your results in the discussion and do not refer to Tables if not necessary.

**AU:** We agree. Paragraph 1 now reads: **(see Line 331)**

“In this study BSI incidence, 30-day all-cause mortality and AMR-bacteria increased over the study period. Surprisingly, the study showed lower 30-day all-cause mortality among patients with BSI caused by AMR bacteria including ESBL, MRSA and VRE without MDR, compared to those with susceptible bacteria. The dominant risk factor for 30-day all-cause mortality associated with BSI was comorbidity, which is in agreement with previous studies [3, 32, 33]. “

**14.** - line 361: I am not sure. At the same time guidelines recommend shorter courses of antibiotic treatment for many indications compared to 10 years ago. If you stick to this argument, please give a reference.

**AU:** Swedish guidelines recommend initially higher doses of antibiotics given more frequently in sepsis and septic shock, based on increased volume of distribution and decreased tissue concentrations. We have added following references: **(see Line 363)**

Nonadherence to antibiotic guidelines in patients admitted to ICU with sepsis is associated with increased mortality: A registry-based, retrospective cohort study**.** Lindberg, De Geer et al 2020.

- **Swedish Association of Infectious Diseases**: Vårdprogram. Sepsis och septisk chock – tidig identifiering och initial handläggning, 2018, <https://infektion.net/wp-content/uploads/2018/06/revision-sepsis-och-septisk-chock-180626.pdf>.
- Kumar A. An alternate pathophysiologic paradigm of sepsis and septic shock: implications for optimizing antimicrobial therapy. Virulence 2014;5:80-97

Thallinger C, Buerger C, Plock N, et al. Effect of severity of sepsis on tissue concentrations of linezolid. J Antimicrobial Chemotherapy 2008;61:173-6

- Roberts JA, Paul SK, Akova, et al. DALI: Defining antibiotic levels in intensive

care unit patients: are current β-lactam antibiotic doses sufficient for critically ill patients? Clin Inf Dis 2014a;58:1072-83

Joukhadar C, Frossard M, Mayer BX, et al. Impaired target site penetration of β-lactams may account for therapeutic failure in patients with septic shock. Crit Care Med 2001:9:385-91

**Reviewer #2: Methods**
**1.** Were all BSIs diagnosed in-hospital or in the community as well? Please specify

**AU:** All microorganisms isolated from outpatient and hospital care were included in the microbiology data and matched with data from the patient care administration system for the BSI analyses **(Lines 130-136)** and we have now added the following text: **(see line 143)**

“All isolated microorganisms from outpatient care and hospital admissions were analyzed at the species level. Species identification and susceptibility testing were performed at the Region’s Department of Clinical Microbiology”.

**2.** What methods do you use for pathogen identification and antimicrobial susceptibility?

**AU:** The following text has been added: **(see Line 144)**

“Matrix-assisted laser desorption ionization time of flight mass spectrometry (MALDI-TOF MS) was used for microbial identification. Clinical susceptibility categories (susceptible, intermediate, and resistant) are defined by cut-offs or breakpoints for different antibiotics and bacteria using the disc diffusion method on Mueller-Hinton agar (MHA). For some bacteria, a complementary E-test was also applied”.

**3.** You defined that the intermediate (I) category was excluded from the resistance analyses. If the numbers are small it may be negligible, but consider adding it to the resistant (R) category.

**AU:** New definitions of S, I and R were introduced 2019 by EUCAST (European Committee on Antimicrobial Susceptibility Testing) as follows:

“*I - Susceptible, increased exposure*: A microorganism is categorised as "Susceptible, Increased exposure*" when there is a high likelihood of therapeutic success because exposure to the agent is increased by adjusting the dosing regimen or by its concentration at the site of infection.”* <http://www.eucast.org/newsiandr/>

**Exposure is a function of how the mode of administration, dose, dosing interval, infusion time, as well as distribution and excretion of the antimicrobial agent will influence the infecting organism at the site of infection.*

For surveillance purposes EUCAST advises against lumping categories, and results should be recorded as S, I and R. If lumping does occur, EUCAST recommends never to lump I + R, only S + I. Furthermore, a new category of Area of Technical Uncertainty (ATU) has been defined and implemented to warn laboratories of uncertain interpretation of antimicrobial susceptibility testing (AST) results. In the old S I R system, the intermediate (I) category included both these isolates with ATU and those needing increased exposure. Thus, only R isolates were included in this study. Thus, we have only included R isolates in this study. In general, EUCAST does not use broad I-groups – normally just 1-2 dilution steps, and only in situations where there are several dosing regimens. For this reason, including or excluding the I-group normally does not make a substantial difference when calculating the R-percentage.

This information has been included in the Methods section as follows: **(see Line 147)**

“Antimicrobial susceptibility clinical breakpoints used during study period were classed as Susceptible (S), Intermediate (I) or Resistant (R) (European Committee on Antimicrobial Susceptibility Testing EUCAST) [22]. The intermediate (I) category was excluded from the resistance analyses based on the new definitions of I performed by EUCAST (<http://www.eucast.org/newsiandr/>). For surveillance purposes EUCAST advices against lumping categories and results should be recorded as S, I and R. If lumping does occur, EUCAST recommends to never lump I + R, only S + I. No major changes in cut-off values for resistance (R), that could have affected susceptibility testing results, were made during the study period”.

**4.** In analyses involving mortality only the first BSI per admission was included. How many patients had recurrent admissions with BSIs? If a significant amount then that introduces bias.

**AU:** In Discussion **(Line 394)** we mention: “However, since only 2% of patients had more than one BSI during the same admission, this limitation could only have had a minor influence on the results. Further, we include the following in the **Flow-chart:** “***9,268 Episodes of BSI consisted of 8,498 patients (patients with ≥3 BSI episodes/year n = 142, 2= n317, 1= n311).”

**AU:** “and mortality” has been added to the text: **(see Line 392)**

“Another limitation was that risk factor and mortality analyses were based on the primary BSI even if a patient admitted for a community-acquired BSI suffered a hospital-acquired BSI during the same admission”.

**5.** From the results I understand that antibiotic consumption data was based on dispensing. Please specify in methods.

**AU:** This has been clarified in Methods. The text now reads: **(see Line 187)**

“All drug statistics were based on sales statistics measured as defined-daily-doses. Data were collected from national drug consumption statistics published by the Swedish e-Health Agency and accessed through a dedicated secure website [30].”

Results
**6.** Add the excluded BSIs and reasons for exclusion.

**AU:** Based on positive blood cultures we excluded microorganisms according to our definitions of BSI and BSI episodes. We have added the following flow-chart describing our study-design in the Methods section **(see Line 136).**

**7.** Data are lacking regarding source of infection, place of acquisition, number of patients (not only number of BSIs)- if available please add

**AU:** The lack of such data has been included as a main limitation of the study: **(see Line 399)**

“A major limitation is that individual patient data on severity of disease, site of infection and appropriate antibiotic treatment were not available in our database”.

**AU:** Between January 2008 and December 2016, a total of 8,498 patients were admitted with a BSI fulfilling the inclusion criteria and subsequently included in the analysis. **(See Line 136: Flow-chart)**

**8.** Antimicrobial resistance- instead of stressing specific bacteria, e.g. Klebsiella oxytoca resistant to PTZ (only 0-3 cases), I would specify general data- quinolone-resistant Enterobacteriaceae, PTZ-resistant Enterobacteriaceae, etc.

**AU:** We have shown the incidence of BSI and antimicrobial resistance per microorganism. The reason for not pooling data from several species was the large differences in incidence over time (*e.g.* E. coli increased 93% and K. oxytoca decreased 5%) and the mechanism of resistance may vary between species.

**AU:** We have added the suggested information regarding Enterobacteriaceae in **Table S12** and **Line 270** in the Results section as shown below:

**S12 Table. Antibiotic resistance in Enterobacteriaceae (2008-2016)**

|  |  |  |  |  |  |  |  |  |  | Regression per 100.000 hospital admissions | | | |
| --- | --- | --- | --- | --- | --- | --- | --- | --- | --- | --- | --- | --- | --- |
|  | **2008** | **2009** | **2010** | **2011** | **2012** | **2013** | **2014** | **2015** | **2016** |  | **AAI %*** | **95% CI** | **p-value** |
| Enterobacteriaceae (n) | 355 | 396 | 525 | 496 | 581 | 582 | 645 | 723 | 600 |  | 49 | (27.42-69.89) | <0.01 |
| Resistance |  |  |  |  |  |  |  |  |  |  |  |  |  |
| Fluoroquinolone (%) | 3.66 | 5.56 | 6.86 | 6.65 | 8.61 | 6.19 | 9.61 | 10.37 | 7.67 |  | 0.8 | (0.21-1.32) | 0.01 |
| PTZ (%) | 2.27 | 1.52 | 1.33 | 1.61 | 3.44 | 2.75 | 1.55 | 4.43 | 3.83 |  | 0.4 | (-0.03-0.75) | 0.07 |
| TMP/SMX (%) | 10.98 | 16.94 | 13.50 | 11.91 | 17.06 | 15.15 | 14.72 | 17.54 | 13.36 |  | 0.3 | (-0.65-1.22) | 0.49 |
| Cephalosporin (%) | 2.54 | 5.05 | 2.67 | 3.63 | 4.65 | 5.15 | 5.12 | 5.67 | 5.17 |  | 0.4 | (0.06-0.75) | 0.03 |
|  |  |  |  |  |  |  |  |  |  |  |  |  |  |
| *AAI = Averageg annual increase % | |  |  |  |  |  |  |  |  |  |  |  |  |

**Line 270:** During the study period we observed significant increases in Fluoroquinolone-resistance (3.7 to 7.7% p = 0.01) and Cephalosporin-resistance (2.5 to 5.2% p = 0.03) amongst Enterobacteriaceae **(S13 Table).**

**9.** Table 2- I would change "univariate analysis" to "characteristics"

**AU:** This has been done. **(see Line 300)**

**10.** Table 3- specifying male and female both is redundant, there are no units for some of the categories (>65- %, charlson score- maybe median (IQR)), for mean (age) add SD.

**AU:** This has been done. **Table 3, Line 313.**

**11.** Table 4- this is the only place that refers to the multivariate analysis for BSI incidence. There is no reference to these results in the text. Also this means that data on patients without BSI had to be extracted and if so, however there is no mention of these patients?

**AU:** We have referred to Table 4 in the Results section, **Line 322.** We do not understand the last question: we calculate ratios, both patients with and without BSI are included.

Discussion
**12.** The result that AMR BSI confers protection against mortality is, as mentioned, very surprising. Explaining this by the observation that they were younger and had less comorbidity is not enough as this is surprising as well. Also, I don’t understand the claim that they were less subject to treatment limitation

**AU:** We agree, this section (Lines 382-387) needed rewriting and we have deleted the following: “~~In the present study, patients with BSI caused by AMR bacteria were younger, had fewer Comorbidities~~ ~~and were less likely to be subjected to treatment limitation as opposed to sick patients with several comorbidities”~~ The text now reads: **(see Line 385)**

“Further studies are needed to explain the lower mortality rate found in this group. In the present study, patients with a BSI caused by AMR bacteria were younger and had less comorbidity. It is possible that people of this age are more exposed to AMR-bacteria because of frequent travel. Furthermore, patients with AMR BSI may receive longer intravenous antibiotic therapy and spend more time in hospital as well, thereby may reducing the chance of recurrent infection. A current lack of resources obliges us to reduce in-hospital times with the result that patients with sensitive bacteria might discharged prematurely. In Sweden, patient with AMR bacteria usually treated by an infectious disease specialist. Infectious disease consultation has been shown to improve outcome in S. aureus sepsis *(”Bai AD, Showler A, et al. Impact of Infectious Disease Consultation on Quality of Care, Mortality, and Length of Stay in Staphylococcus aureus Bacteremia: Results From a Large Multicenter Cohort Study, 2015)* and this may also have influenced the treatment of BSI caused by AMR bacteria of other species including ESBL-E. coli which was the most prevalent AMR bacteria found in this study. Improved care due to involvement of an ID specialist may explanation for the better outcome among patients with AMR bacteria. This could well be the explain the lower mortality rate found in this group [48, 51-53]. This warrants further study and we are planning a case-control study with extensive data on patients and bacteria, including virulence factors, in order to gain a better insight into why patients with ESBL-E. coli BSI have a better outcome.”

Grammar
**13.** Line 89- pathogens instead of agents

**AU:** This has been done: **(see Line 89)**

“Enterobacteriaceae resistant to fluoroquinolones followed by Enterobacteriaceae resistant to 3^rd^ generation cephalosporins are probably the most frequently encountered and clinically important antimicrobial-resistant pathogens in Sweden today [8-10]. “

**14.** Line 126- in 2000-2013

**AU:** This has been done. **(see Line 126)**

“Data were extracted from the Region Östergötland BSI register as in a previous study performed in 2000-2013.”

**Reviewer #3:**

I don't have many comments. I think this was a good piece of research, well-written with appropriate statistics. My only suggestions is that the Definitions section should come in an appendix rather than in the middle of the manuscript and the thought that the reason that the mortality burden of AMR was low was perhaps due to the restricted use of antibiotics in primary care in Sweden. The authors may want to suggest this.

**AU:** Thank you very much. We believe that the Definitions section is very important for the readers, and if the Editor has nothing against it, we suggest that the Definitions section remains in the manuscript. We agree on the importance of restricted use of antibiotics in primary care in Sweden and have mentioned this in **Line 375:**

“Only 2.6% of all BSIs were caused by AMR bacteria. The restricted use of systemic antibiotics in the Swedish primary healthcare system and in animals, probably explains why we have a relatively low prevalence of AMR bacteria compared to other European countries.”

**Reviewer #4:**

The authors performed a nine-year observational retrospective study aimed at describing the epidemiology of BSIs in a county in South-East Sweden. Data on BSI episodes were gathered from the microbiology laboratory database and linked to electronic health records available for the same hospitalization. At the same time the authors describe the trend of antimicrobial consumption in the same hospitals by analyzing DDDs/patient days and consumption in the outpatient setting by analyzing DDs/inhabitants. The study shows a rising trend in the incidence of BSIs and also a rising 30-day mortality rate in patients diagnosed with bacteremia. In-hospital antimicrobial consumption is also increasing, probably as the consequence of the increasing age and comorbidities of admitted patients. The authors already published part of this dataset, showing the relationship between comorbidities and mortality in bacteremic patients. In this new study, they analyzed a larger cohort adding data on antimicrobial resistance and antimicrobial consumption.

**1.** My main concern with this study is the unclear study design. When dealing with large databases, it is very common to find significant results and infer causal relationship from these results. However, this approach should generally be avoided if there is no a pre-defined/biologically plausible hypothesis grounding the analysis. In this case, for example, the authors found a significant association between comorbidities and 30-day mortality, proving a biologically plausible hypothesis (already present in previous results from the same cohort). However, when coming to AMR, the analysis shows that AMR has a significant protective effect, even after correcting with comorbidities and age. Unfortunately, with the available data this cannot be explained and proves the difficulty of dealing with this type of data. When testing risk factors for mortality in BSIs we should probably not accept analyses that cannot include relevant patient-level data, such as antibiotic therapy and appropriate measures of infection severity.

**AU:** We agree that since this a retrospective observational study, based on many BSIs, you cannot infer causal relationships regarding *e.g.* observed risk factors for mortality and BSI. However, the study design is useful for population-based surveillance of BSIs including patient age and comorbidity data and microbiology data on causative pathogens and their susceptibility patterns. Although we lack data on competing mortality events, the study design allows assessment of the association between species and mortality which showed higher mortality due to S. aureus BSIs than E. coli BSIs. This was in agreement with our hypothesis and with other studies *(Skogberg K, et al. P Population-based burden of bloodstream infections in Finland).* Our hypothesis regarding burden of resistance was rejected and in fact we observed lower mortality among patients with BSIs caused by ESBL-E. coli compared to fully susceptible E. coli! But data are data, they must be reported and critically analyzed as we point out in the discussion. We do not conclude that AMR has a protective effect against mortality, but the findings in this study has led to a new case-control study on patients with urosepsis caused by ESBL-E. coli vs fully susceptible E. coli. In order to better explain the lower mortality among ESBL-E. coli BSIs, we will assess virulence factors, appropriate antibiotic treatment, severity of illness etc.

**2.** Few other comment on the text follows:
Abstract: the study-design should be mentioned in the abstract and in the methods it should be clearly stated how the data collection was performed and how the study was planned (retrospective cohort from electronic records..).

**AU:** Thank you. We have added in the Abstract and Methods section that this is a retrospective cohort study based on data from electronic records. The text now reads: **(see Lines 50 and 123)**

**Line 50:** “A 9-year (2008-2016) retrospective cohort study from electronic records of episodes of bacteremia amongst hospitalized patients in the county of Östergötland, Sweden, was conducted”

**Line 123:** “Study design: A retrospective cohort study on data from electronic records describing and analyzing the incidence and 30-day all-cause mortality of culture-confirmed BSI in the Region of Östergötland, Sweden, between January 1, 2008 and December 31, 2016.”

**3.** AMR/MDR definition: definition used is quite tricky because MRSA should be always considered MDR. It is difficult to imagine that approximately 1/3 of ESBLs are resistant to less than 3 classes (so not falling under the MDR definition). I would suggest reconsidering this categorization dividing pathogens according to their phenotypic resistance, rather than number of classes (~~FQ-R E~~. coli; ESBL-Ent; MRSA, VRE…).

**AU:** When designing this study we decided to use the generally accepted definition of multidrug resistance (MDR) *i.e.* non-susceptibility to at least 1 agent in ≥3 antimicrobial classes (Falagas, J Med Microbiol, 2006 and Magiorakos, A.P., et al., Clin Microbiol Infect, used by WHO, ECDC)

However, applying this MDR definition to our data excludes a significant number of ESBL-producing Enterobacteriaceae not fulfilling the MDR criteria thereby underestimating the frequency of AMR. We thus report not only MDR but also AMR without multidrug resistance (MRSA, ESBL-producing Enterobacteriaceae, vancomycin-resistant enterococci not fulfilling the MDR criteria). As seen in Table 3, 76% of AMR-BSIs were caused by MDR-BSIs and 24% were AMR-BSIs without multidrug resistance including ESBL, MRSA and VRE. Presentation of data in this paper enables comparison with ECDC data on the frequency of 3rd generation Cephalosporin-resistant Enterobacteriaceae (ESBL phenotype), MRSA, VRE and MDR E. coli/Klebsiella.AMR and MDR definitions are included in Methods **(see Lines 169-173 in Methods section)** and we have added a comment in Discussion. The text now reads: **(see Line 381)**

“In the design of this study we decided to use the generally accepted definition of multidrug resistance (MDR) *i.e.* non-susceptibility to at least 1 agent in ≥3 antimicrobial classes (Falagas, J Med Microbiol, 2006 and Magiorakos, A.P. *et al*, Clin Microbiol Infect)

However, application of this MDR definition to our data would excludes a significant number of MRSA, ESBL-producing Enterobacteriaceae not fulfilling the MDR criteria thereby underestimating the frequency of AMR. Thus we report MDR but also AMR without multidrug resistance (MRSA, ESBL-producing Enterobacteriaceae, vancomycin-resistant enterococci not fulfilling the MDR criteria).”

**4.** Discussion:
-Increasing incidence and blood cultures: in the discussion the authors state that the increase number of BSIs is probably not due to the growing number of patients tested because the proportion of positive blood cultures remain the same. I would advise to report somewhere the overall number of blood cultures performed, rather than the proportion to better exclude this confounder.

**AU:** The total number of blood cultures is shown in **Table S5.**

**5.** -Line 379: association between AMR and antimicrobial consumption, sanitation, food safety is a very complex issue. I would advise rephrasing as ‘…these are all concomitant factors that could explain the low level of AMR rates in Sweden’.

**AU:** This has been done: **(see Line 379)**

“Other concomitant factors that could explain the low level of AMR rates in Sweden include high degree of food safety, improved hygiene and infection prevention measures, and meticulous sanitation.”

**6**. -Line 386: what do the author mean with treatment limitation? The association with AMR remains significant even if adjusting per age and comorbidities, so they probably should not be considered as relevant factors.
**AU:** We agree, this section (line 382-387) needs rewriting and we have deleted the following Line 386. “~~In the present study, patients with BSI caused by AMR bacteria were younger, had fewer Comorbidities~~ ~~and were less likely to be subjected to treatment limitation as opposed to sick patients with several comorbidities”~~ The text now reads: **(see Line 386)**

“Further studies are needed to explain the lower mortality rate found in this group. In the present study, patients with a BSI caused by AMR bacteria were younger and had less comorbidity. It is possible that people of this age are more exposed to AMR-bacteria because of frequent travel. Furthermore, patients with AMR BSI may receive longer intravenous antibiotic therapy and spend more time in hospital as well, thereby may reducing the chance of recurrent infection. A current lack of resources obliges us to reduce in-hospital times with the result that patients with sensitive bacteria might discharged prematurely. In Sweden, patient with AMR bacteria usually treated by an infectious disease specialist. Infectious disease consultation has been shown to improve outcome in S. aureus sepsis *(”Bai AD, Showler A, et al. Impact of Infectious Disease Consultation on Quality of Care, Mortality, and Length of Stay in Staphylococcus aureus Bacteremia: Results From a Large Multicenter Cohort Study, 2015)* and this may also have influenced the treatment of BSI caused by AMR bacteria of other species including ESBL-E. coli which was the most prevalent AMR bacteria found in this study. Improved care due to involvement of an ID specialist may explanation for the better outcome among patients with AMR bacteria. This could well be the explain the lower mortality rate found in this group [48, 51-53]. This warrants further study and we are planning a case-control study with extensive data on patients and bacteria, including virulence factors, in order to gain a better insight into why patients with ESBL-E. coli BSI have a better outcome.”

**Reviewer #5:**

This study investigated the risk factors for 30-day mortality among hospitalized bacteraemia patients in a county in Sweden over a nine-year period, using routinely-collected linked data and taking into consideration antibiotic susceptibility. Overall, I read this article with interest and appreciate how much work the authors have done and how much data they have provided. I do, however, think amendments and additional clarification need to be provided before this article can be considered for publication.

Main amendments:
**1.** Line 147 – Why were the intermediate isolates excluded? In many other studies “intermediate” and “resistant” have been combined to the category of “non-susceptible” and compared with “susceptible”. It could be a useful sensitivity analysis to investigate whether including these isolates in the “non-susceptible” category alters the results significantly. Alternatively, quantifying how many intermediate isolates were excluded and what the rationale behind this decision was would be helpful.

**AU:** New definitions of S, I and R were introduced 2019 by EUCAST (European Committee on Antimicrobial Susceptibility Testing) as follows:

“*I - Susceptible, increased exposure*: A microorganism is categorised as "Susceptible, Increased exposure*" when there is a high likelihood of therapeutic success because exposure to the agent is increased by adjusting the dosing regimen or by its concentration at the site of infection.”* <http://www.eucast.org/newsiandr/>

**Exposure is a function of how the mode of administration, dose, dosing interval, infusion time, as well as distribution and excretion of the antimicrobial agent will influence the infecting organism at the site of infection.*

For surveillance purposes EUCAST advises against lumping categories, and results should be recorded as S, I and R. If lumping does occur, EUCAST recommends never to lump I + R, only S + I. Furthermore, a new category of Area of Technical Uncertainty (ATU) has been defined and implemented to warn laboratories of uncertain interpretation of antimicrobial susceptibility testing (AST) results. In the old S I R system, the intermediate (I) category included both these isolates with ATU and those needing increased exposure. Thus, only R isolates were included in this study. Thus, we have only included R isolates in this study. In general, EUCAST does not use broad I-groups – normally just 1-2 dilution steps, and only in situations where there are several dosing regimens. For this reason, including or excluding the I-group normally does not make a substantial difference when calculating the R-percentage.

This information has been included in the Methods section as follows: **(see Line 147)**

“Antimicrobial susceptibility clinical breakpoints used during study period were classed as Susceptible (S), Intermediate (I) or Resistant (R) (European Committee on Antimicrobial Susceptibility Testing EUCAST) [22]. The intermediate (I) category was excluded from the resistance analyses based on the new definitions of I performed by EUCAST (<http://www.eucast.org/newsiandr/>). For surveillance purposes EUCAST advices against lumping categories and results should be recorded as S, I and R. If lumping does occur, EUCAST recommends to never lump I + R, only S + I. No major changes in cut-off values for resistance (R), that could have affected susceptibility testing results, were made during the study period”.

**2.** Lines 159-161 and lines 166-168 – How did the authors handle non-identical antibiograms? Was the most resistant antibiogram chosen (standard practice)? What about polymicrobial blood cultures?

**AU:** The following information has been included in the Methods section:

Repeat isolate: Culture of same species with identical resistance pattern isolated during the same admission period (from admission until hospital discharge). Only initial bacteria or yeast isolates were considered; thus repeat isolates of the same species with the same antibiogram from the same patient were excluded. Change in antibiogram was defined as a change in degree of susceptibility (S and R or R and S). Number of polymicrobial blood cultures have been added in the flow-chart.

For example, if a patient had a fully susceptible E. coli in the initial blood culture and there was growth of E. coli resistant to FQ in a second blood culture a few days later, the FQ-resistant E. coli blood culture was defined as a new BSI and included in the analysis.

**3.** Lines 171-173 – Why was AMR not simply defined as any culture resistant to any of the antibiotics included in the standard panel? Using the definition outlined by the authors, were some antibiotic-resistance profiles missed?

**AU:** In Sweden we have low occurrence of resistance and most of the resistance reported here was clinically irrelevant for outcome. That is why we use a broader definition of AMR in some of the analyses. In Table S6 we present resistance to all antibiotics included in the standard panel.

**4.** Lines 174-179 – How was the new BSI episode definition devised (references, expert opinion etc.)? What is the rationale behind only using the first BSI episode within a hospitalization? I would imagine that patients with more than one BSI episode (defined as being caused by different aetiogical agents) would be at a higher risk of dying within 30 days?

**AU:** When analyzing 30-day all-cause mortality we use the first day of a BSI episode (positive blood culture). If a patient had a fully susceptible pathogen in the initial blood culture and there was growth of the same species a few days later but resistant to one or more drugs it was defined as a new BSI and included in the analysis. Regarding repeated BSI episodes, we have mentioned this in the discussion (only 2% of patients that had more than one BSI during the same admission), this limitation could only have had a minor influence on the results

**5.** Lines 182-183 – Outlining the conditions that make up the Charlson Comorbidity Index would be useful, as well as how the Index is calculated and whether the CCI was the only measure of comorbidity used or whether comorbidities were looked at separately.

**AU:** This has been done. We have added the following text to the Definitions section: (see **Line 182** and the newly added **Table S13** with CCI and how this was calculated). We have reported CCI and numbers of comorbidities because it could be misleading to only calculate CCI with focus on severity. Numbers of comorbidities, multi-comorbidity may have an important clinical impact for the patient in clinical management.

“Comorbidity based on the International Classification of Diseases (ICD) was extracted from the Patient Care Administration System from the day of admission and 24 months prior to that (ICD-10-CA and the updated Charlson Score Index) [27-29]. (**Table S13**)”

**6.** Definitions – Please outline whether mortality was 30-day all-cause mortality following a BSI episode and/or how mortality was attributed to the BSI. If the measure was 30-day all-cause mortality following a BSI, please update the wording of this throughout the manuscript.

**AU:** This has been done. We have changed “30-day mortality” to “30-day all-cause mortality” in the manuscript.

**7.** Lines 234-242 – It would possibly be more informative to report on the relative proportions of different bacterial species causing BSI between 2008 and 2016 as opposed to the increase for each pathogen. Reporting pathogen increase could simply be related to an overall increase in BSI incidence, whereas reporting proportions of aetiological agents could give an indication of changes in the most prevalent species/pathogenicity etc.

**AU:** We have reported each bacterial species separately, because of the large differences in numbers of BSI per pathogen. For instance, the number of Escherichia coli BSIs in 2016 was n390, Acinetobacter baumannii was n3 and Hemophilus influenzae was n4. That is why we did not pool them. Linear Regression per 100.000 hospital admissions was performed to analyze the average annual increase rather than an overall increase in BSI.

**8.** Table 3 – Why are there two measures of age? Why is comorbidity measured in three separate ways (individual conditions, number of comorbidities and CCI)? How do the authors know that the individual comorbidities were not the cause of death?

**AU:** We have adjusted age **(see Line 314**). We chose to specify comorbidity in each group (survivor and non-survivor) and to compare this. We also wanted to see if difference in the number comorbidities between groups had an effect. In the analysis of CCI, specific comorbidities gave different points in first hand based on severity **(Table S13**). Numbers of comorbidities, multi-comorbidity may have an important clinically impact for the patient in clinical management. We do not know if the individual comorbidities were the cause of death.

**9.** Table 4 – Why was number of comorbidities chosen to be part of the adjusted model instead of CCI? CCI showed a significant relationship with the outcome in the univariate analysis and I would argue is a superior measure of overall illness and frailty as it applies different weightings of severity to different conditions to compile an overall composite measure of multi-comorbidity.

**AU:** We agree with this comment and could have chosen CCI in our multivariable analysis. We chose “number of comorbidities” because this showed a significant relationship with outcome in the univariate analysis, we think it have an important clinically impact for the patient in clinical management.

**10.** Lines 359-361 – Is the proportion of patients with multiple comorbidities increasing over time after adjusting for changes in demographics (age) or is this simply due to an aging population? Perhaps worth discussing a bit more.

**AU:** Age did not increase significantly over the study period (from 69 to 70 years see Table S5). Comorbidity analysis was based on comorbidity per 100.000 hospital admissions and year. Number of hospital admissions appeared to be stable (Table S5). The increase in the number of comorbidities probably depends on better health, oncology treatment etc. Patients with several comorbidities live longer today than previously.

**11.** Lines 361-362 – Are “modern guidelines” truly recommending “higher and more frequent doses of antibiotics” in Sweden? This is in contradiction with most prescribing guidelines in other high-income settings as antibiotics are recommended for fewer conditions, delayed antibiotic prescribing or “wait and see” approaches are more frequently advised and shorter antibiotic durations are more frequently recommended as first line treatment.

**AU:** Swedish guidelines recommend initially higher doses of antibiotics given more frequently in sepsis and septic shock, based on increased volume of distribution and decreased tissue concentrations. We have added following references: **(see Line 363)**

Nonadherence to antibiotic guidelines in patients admitted to ICU with sepsis is associated with increased mortality: A registry-based, retrospective cohort study. Lindberg, De Geer et al 2020.

- **Swedish Association of Infectious Diseases**: Vårdprogram. Sepsis och septisk chock – tidig identifiering och initial handläggning, 2018, <https://infektion.net/wp-content/uploads/2018/06/revision-sepsis-och-septisk-chock-180626.pdf>.
- Kumar A. An alternate pathophysiologic paradigm of sepsis and septic shock: implications for optimizing antimicrobial therapy. Virulence 2014;5:80-97

Thallinger C, Buerger C, Plock N, et al. Effect of severity of sepsis on tissue concentrations of linezolid. J Antimicrobial Chemotherapy 2008;61:173-6

- Roberts JA, Paul SK, Akova, et al. DALI: Defining antibiotic levels in intensive

care unit patients: are current β-lactam antibiotic doses sufficient for critically ill patients? Clin Inf Dis 2014a;58:1072-83

Joukhadar C, Frossard M, Mayer BX, et al. Impaired target site penetration of β-lactams may account for therapeutic failure in patients with septic shock. Crit Care Med 2001:9:385-91

**12.** Line 375 – Could the authors please provide a bit more context around the “restricted use of systemic antibiotics” in Sweden? What kinds of policies have been implemented to achieve this?

**AU:** In 1995 the Swedish strategic program against antibiotic resistance (STRAMA) began. This works at both local and national levels, and has the following ongoing strategies:

1. active monitoring of antibiotic use, providing data for informed decision-making; 2. national guidelines for antibiotic prescription; 3. local, national and global antibiotic resistance surveillance to signal warnings and promote action; 4. tracking resistance trends; 5. meticulous infection control to limit spread of resistance; and 6. better communication to increase our awareness of the need for action and behavioral change. A key element in the achievement of long-term changes has been the bottom-up approach, including working closely with prescribers at the local level. Our work in this field and the lessons learnt, as described here, provides a basis upon which other countries can develop their own national plan of action against antibiotic resistance.

Reference:

**Mölstad, Löfmark et al.** Lessons learnt during 20 years of the Swedish strategic programme against antibiotic resistance. 2017. Bull World Health Organ

**13.** Lines 382-388 – As this is quite a surprising result which is not in line with previous comparable studies, I believe this requires more discussion as to why this particular patient group (younger, less ill patients) had a higher rate of MDR infections, leading to the effect on morbidity. Could there be factors related to travel? Better antibiotic stewardship in long-term care facilities and primary care making the elderly less likely to receive antibiotics (although this seems unlikely, but perhaps possible in Sweden)? More discussion around possible contributing factors would be welcome.

**AU:** We agree, this section (line 382-387) needs rewriting and we have deleted the following Line 386. “~~In the present study, patients with BSI caused by AMR bacteria were younger, had fewer Comorbidities~~ ~~and were less likely to be subjected to treatment limitation as opposed to sick patients with several comorbidities”~~ The text now reads: **(see Line 386)**

“Further studies are needed to explain the lower mortality rate found in this group. In the present study, patients with a BSI caused by AMR bacteria were younger and had less comorbidity. It is possible that people of this age are more exposed to AMR-bacteria because of frequent travel. Furthermore, patients with AMR BSI may receive longer intravenous antibiotic therapy and spend more time in hospital as well, thereby may reducing the chance of recurrent infection. A current lack of resources obliges us to reduce in-hospital times with the result that patients with sensitive bacteria might discharged prematurely. In Sweden, patient with AMR bacteria usually treated by an infectious disease specialist. Infectious disease consultation has been shown to improve outcome in S. aureus sepsis *(”Bai AD, Showler A, et al. Impact of Infectious Disease Consultation on Quality of Care, Mortality, and Length of Stay in Staphylococcus aureus Bacteremia: Results From a Large Multicenter Cohort Study, 2015)* and this may also have influenced the treatment of BSI caused by AMR bacteria of other species including ESBL-E. coli which was the most prevalent AMR bacteria found in this study. Improved care due to involvement of an ID specialist may explanation for the better outcome among patients with AMR bacteria. This could well be the explain the lower mortality rate found in this group [48, 51-53]. This warrants further study and we are planning a case-control study with extensive data on patients and bacteria, including virulence factors, in order to gain a better insight into why patients with ESBL-E. coli BSI have a better outcome.”

**14.** Lines 404-406 – This is indeed a major limitation, the possible implications of which should be discussed and explored further.
**AU:** We agree and mention this as a limitation. We have also changed “30-day mortality” to “30-day all-cause mortality” in the text. Another study-design would have been necessary to analyze cause of death, and this was impossible in the present study because of the enormous volume of data.

Smaller amendments:
**15.** Line 78 – Define BSI in the first instance

**AU:** This has been done. The text now reads: **(see Line 78)**

“The high burden of bloodstream infection (BSI) and increasing prevalence of BSI caused by antimicrobial-resistant (AMR) and multidrug-resistant (MDR) bacteria is a serious threat to global public health [1-6].”

**16.** Line 84 – “…has not increased significantly in the last…”

**AU:** This has been done. The text now reads: **(see Line 84)**

“MRSA causes only 1.2 % of all *Staphylococcus aureus* BSIs in Sweden and has not increased significantly in the last 20 years.”

**17.** Line 86 – Is this increase in Sweden in 2017?

**AU:** Yes

“However, *Escherichia coli* resistant to 3^rd^ generation cephalosporins, usually producing extended-spectrum beta-lactamases (ESBL), increased from 5.6% in 2014 to 7.4%

in 2017.”

**18.** Line 88 – Please avoid the use of “probably”

**AU:** This has been done. The text now reads: **(See Line 88)**

“Enterobacteriaceae resistant to third-generation cephalosporins are one of the most common ~~probably the most frequently encountered~~ and clinically important antimicrobial-resistant pathogens in Sweden today [8-10].”

**19.** Line 94 – “Since antimicrobial-resistant organisms have become…”

AU: This has been done. The text now reads: **(see Line 94)**

“Since antimicrobial-resistant organisms have become more prevalent in most countries, the choice of appropriate antibiotic treatment has become increasingly challenging.”

**20.** Line 108 – Other information systems such as? Please specify

**AU:** The text has been changed. Östergötland region, with its catchment population of approximately 450,000 inhabitants (5% of the Swedish population), is served by four hospitals.

The region has developed a database cross-linking microbiological data, mortality data and data from the patient care administration system. The text now reads: **(see Line 108)**

“..is served by four hospitals and has developed a database cross-linking systems providing microbiological data and mortality data with data from the patient care administration system.”

**21.** Line 123 – I would call this a retrospective cohort study – while the authors do present descriptive statistics, a comparative approach is taken when modelling mortality outcomes.

AU: Thank you. We have added in the Abstract and Methods section that this is a retrospective cohort study based on data from electronic records. The text now reads: **(see Lines 50 and 123)**

**Line 50:** “A 9-year (2008-2016) retrospective cohort study from electronic records of episodes of bacteremia amongst hospitalized patients in the county of Östergötland, Sweden, was conducted”

**Line 123:** “Study design: A retrospective cohort study on data from electronic records describing and analyzing the incidence and 30-day all-cause mortality of culture-confirmed BSI in the Region of Östergötland, Sweden, between January 1, 2008 and December 31, 2016.”

**22.** Study design – It would be helpful to include the patient characteristics defining the cohort (age, gender, any other inclusion criteria besides just the region and the clinical diagnosis)

**AU:** In the Methods section we describe the analysis of all positive blood culture in the adult population (≥18 yrs) of a Region in south-east Sweden between 2008 and 2016. In Table S5, data on median age, hospital admission, population of Östergötland, and BSI are given on a yearly basis. Gender is reported in Table 3, and inclusion/exclusions criteria are defined in Methods. We have added a flow-chart to the manuscript to clarify this.

**23.** Lines 192-193 – It is unclear as to whether the systemic antibiotics analysed were both community and hospital or just hospital. Is it possible to differentiate this within the registry so as to accurately define the numerator? Was antibiotic consumption aggregated by the region – please clarify?

**AU:** This is made clear in the Methods section. The text now reads: (see Lines 187 and 189, see also Table S7-S9 where we report the numerator and Lines 251-266 in the manuscript)

**Line 187:** “All drug statistics were based on sales statistics measured as defined-daily-doses. Data were collected from national drug consumption statistics published by the Swedish e-Health Agency and accessed through a dedicated secure website [30].”

**Line 189:** “Consumption of systemic antibiotics were analyzed both in outpatient care and hospital (defined either as J01 in the ATC-code for international comparison, or J01, excluding J01XX05 methenamine, as the national Swedish quality indicator) in both outpatient care and hospital care was measured as defined-daily-doses (DDD) per 1,000 inhabitants and day. Use of systemic antibiotics in hospital was measured as DDD using hospital admission or hospital days as the denominator [30, 31].”

**24.** Line 220 – Please remove “approximately”

AU: This has been done. The text now reads **(See Line 220):**

“A total of 9,268 BSIs fulfilling the inclusion criteria were recorded between January 2008 and December 2016 and subsequently analyzed (S12 Figure). 46% (n=4,242) of the BSI-episodes represented by female and the median age of all patients with a positive blood culture increased from 69 to 70 years (p=0.01).”

**25.** Lines 221-222 – Why not report on the median age over all the years of the study?

AU: We have reported the median age/year in **Table S5.**

**26.** Line 223 – Please refer to Table S4 and include percentages in the table.

AU: This has been done. We have re-analyzed and included percentages in Table S4.. The text now reads: **(see Line 226)**

“The most common agents causing BSI were Gram-negative bacteria (50%), Gram-positive bacteria (38%) other bacteria (8%) and *Candida* spp. (4%). The incidence of BSI increased by 66% from 973 in 2008 to 1,610 in 2016 per 100,000 hospital admissions with an average annual increase of 78 BSI episodes per 100,000 hospital admissions during the study period (p<0.01) (Figure S1, Table S4 and Table S5).”

**27.** Line 243 – Is this the overall proportion of hospital admissions due to any cause or just BSI?

**AU:** Yes: overall proportion of hospital admissions due to any cause.

**28.** Lines 244-247 – This sentence seems to contradict itself, please clarify

**AU:** We have changed the text which now reads: (see Line 246)

“The proportion of hospital admissions during which a positive blood culture was found increased from 1.1 to 1.8% (p<0.01), though there was a minor decrease in the proportion of blood cultures that were positive per total number of blood cultures (from 10.1 to 9.2%, (p = 0.11)) (Table S5).”

**29.** Lines 254-255 – This sentence seems to contradict the previous sentence (both reporting hospital prescribing of systemic antibiotics but with different rates), please clarify

**AU:** 17% = DDD/TIND (1,000 inhabitants and day) 48% = DDD/1,000 hospital days. Both are included to show the difference depending on the denominator, since both have been used in previous studies.

**30.** Antimicrobial resistance – It seems slightly sporadic which trends the authors chose to highlight - were they the largest changes, the most important drug-bug combinations clinically in the region etc.? Table S6 is unclear with respect to percentages and counts, please consider making this table easier to interpret

**AU:** We report significant antimicrobial resistance trends. If choices seem sporadic it is because of few cases and the clinical relevance is difficult to judge. We do, however, report BSIs due to AMR and MDR. In Table S6, we report raw data to enhance the reproducibility of our results.

**31.** Lines 286-288 – This breakdown for 30-mortality is slightly confusing, was ESBL-E not included in AMR BSI? Or was AMR BSI not including ESBL-E or MDR-E? If so, why?

**AU:** ESBL-E was included in the AMR BSI group. In the Methods section under “definitions”, Line 171, reads: “Antimicrobial Resistance (AMR): MDR bacteria and MRSA, ESBL-producing Enterobacteriaceae, vancomycin-resistant enterococci not fulfilling the MDR criteria were defined as antimicrobial-resistant (AMR) bacteria”

And in the Results section Lines 282-288: “A total of 245 AMR BSIs were observed, and among those 185 were caused by MDR bacteria (ESBL *E. coli* (ESBL-E) (n = 74)……. and non-MDR bacteria (n = 60), (ESBL-E (n = 48)”.

**32.** Table 1 – This table is cut off in my PDF and therefore cannot be properly interpreted

**AU:** We have tried to improve the text and illustrations in order to show data better supporting the conclusions.

**33.** Table 2 – This appears to be presenting baseline characteristics, not the results of univariate analyses, please amend

**AU:** This has been done, **(see Line 300).**

**34.** Line 318 – Was age a year-on-year change? If so, I would suggest using a categorical variable for age instead in order to report a more meaningful increase in risk

**AU:** The main reason for performing multivariable analysis was to check that the RR for comorbidities did not change when adjusted for age and gender. The original variable did not change.

**35.** Line 339 – Only one factor is mentioned, are there others?

**AU:** We have changed the text that now reads: **(see Line 339)**

“There are several factors that could explain the increase in the annual incidence of BSI up to 2013, including the increase in the number of blood cultures taken per hospital admission; a direct result of increased compliance with the principle of taking blood cultures before starting antibiotic treatment. However, the proportion of positive blood cultures did not change significantly over the study period. If potential areas for improvement are to be found, other possible explanations for the observed increase in BSI must be studied, both in previously healthy patients and those with comorbidities.”

**36.** Line 401 – “…association between antibiotic use and antibiotic-resistant infection at the patient-level…”

**AU:** We here stress the fact that it was impossible to obtain individual patient data from our database.

In The Discussion the text reads **(See Line 399):**

“A major limitation is that individual patient data on severity of disease, site of infection and appropriate antibiotic treatment were not available in our database. We were thus unable to assess any association between antibiotic use and risk for antibiotic-resistant infection, or between specific empirical regimens and outcome.”

6. PLOS authors have the option to publish the peer review history of their article ([what does this mean?](https://journals.plos.org/plosone/s/editorial-and-peer-review-process#loc-peer-review-history)) If published, this will include your full peer review and any attached files.


If you choose “no”, your identity will remain anonymous but your review may still be made public.


**Do you want your identity to be public for this peer review?** For information about this choice, including consent withdrawal, please see our [Privacy Policy](https://www.plos.org/privacy-policy).

Reviewer #1: No

Reviewer #2: No

Reviewer #3: Yes: Brian Bell

Reviewer #4: Yes: Elena Carrara

Reviewer #5: No


[NOTE: If reviewer comments were submitted as an attachment file, they will be attached to this email and accessible via the submission site. Please log into your account, locate the manuscript record, and check for the action link "View Attachments". If this link does not appear, there are no attachment files to be viewed.]


While revising your submission, please upload your figure files to the Preflight Analysis and Conversion Engine (PACE) digital diagnostic tool, <https://pacev2.apexcovantage.com/>. PACE helps ensure that figures meet PLOS requirements. To use PACE, you must first register as a user. Registration is free. Then, login and navigate to the UPLOAD tab, where you will find detailed instructions on how to use the tool. If you encounter any issues or have any questions when using PACE, please email us at [figures@plos.org](mailto:figures@plos.org). Please note that Supporting Information files do not need this step.
